# An m^5^C-associated NSUN5–YBX1–CENPF programme marks melanoma cell states and immune features

**DOI:** 10.3389/fimmu.2026.1952519

**Published:** 2026-10-16

**Authors:** Aimei Liu, Kun Yuan, Sha Yi, Hanxiao Mao, Xinyu Xie, Shiyang Yin, Haonan Fan, Ailin Zhang, Xixu Liu, Yifu Hu, Aiguo Xu, Wei Chen, Yuanmin He

**Affiliations:** 1 Skin Structure and Function Key Laboratory of Luzhou, Department of Dermatology, The Affiliated Hospital of Southwest Medical University, Luzhou, Sichuan, China; 2 Department of Dermatology, The Second Affiliated Hospital of Chengdu Medical College, China National Nuclear Corporation 416 Hospital, Chengdu, Sichuan, China; 3 Clinical Medical College, Southwest Medical University, Luzhou, Sichuan, China; 4 Department of Dermatology, Chengdu Integrated TCM & Western Medicine Hospital, Affiliated Hospital of Chengdu University of Traditional Chinese Medicine, Chengdu, Sichuan, China

**Keywords:** drug repurposing, melanoma, NSUN5–YBX1–CENPF programme, RNA m^5^C, single-cell transcriptomics, tumour immunity

## Abstract

**Background:**

RNA 5-methylcytosine (m^5^C)-associated programmes may help characterise melanoma cell states and their immune context. We investigated an NSUN5–YBX1–CENPF (NYC) programme using patient-matched sequencing, experimental perturbation and multiscale transcriptomic analyses.

**Methods:**

We analysed m^5^C-MeRIP-seq and matched Input RNA data from three melanoma–adjacent-normal patient pairs, using a paired edgeR interaction model for regional IP enrichment. Tissue and cell experiments included NSUN5 knockdown, CENPF-targeted MeRIP–qPCR and western blotting. Clinical analyses considered proliferation and sampling site. Trajectory and regulatory inference were restricted to malignant cells from GSE215120, with patient-level summaries. Treatment-associated changes were examined in 43 paired patients from GSE91061. Computational perturbation and transcriptomic signature reversal prioritised candidate regulators and compounds.

**Results:**

Two CENPF regions showed increased relative IP enrichment after Input adjustment (4.97- and 5.85-fold; BH q = 0.0457 and 0.0074). NSUN5 knockdown reduced CENPF m^5^C enrichment and protein abundance. NYC was associated with proliferation; its survival association did not persist after proliferation adjustment. Within malignant cells, NYC-High retained an association with an immune-resistance expression programme after G2M/E2F and depth adjustment (nine patients; BH q = 0.0156). Regulatory analyses prioritised MLX, whose simulated knockout and overexpression produced opposing reference-flow alignments (BH q = 0.0134 and 0.0201). MLX regulon activity was associated with glycolysis and oxidative phosphorylation after adjustment. In GSE91061, responders showed a greater treatment-associated NYC decrease than non-responders (mean change difference −0.209; permutation P = 0.0138). Drug screening generated candidates for experimental testing.

**Conclusions:**

NYC provides an m^5^C-associated framework connecting patient-derived molecular observations to malignant-cell states and immune features. YBX1 remains a candidate reader, and regulatory, treatment-associated and drug-prioritisation findings do not establish a complete causal pathway or a clinically validated predictive biomarker.

## 1 Introduction

Cutaneous melanoma has a strikingly stage-dependent clinical course: early-stage disease can often be durably controlled by complete surgical excision, whereas advanced melanoma continues to impose a substantial mortality burden (1, 2). Immune checkpoint blockade targeting CTLA-4 and PD-1 has markedly prolonged survival in a subset of patients with advanced disease, with treatment strategies evolving from monotherapy to combination regimens (3–5). Long-term follow-up has confirmed the durability of benefit from combined immunotherapy, whilst dual BRAF/MEK inhibition has reshaped the therapeutic landscape for patients with BRAF-mutant melanoma (6, 7). Nevertheless, primary resistance, acquired resistance and treatment-driven co-evolution of tumour cells and their microenvironment continue to preclude curative outcomes for many patients (8, 9). Molecular classification by The Cancer Genome Atlas further revealed extensive heterogeneity in melanoma driver alterations and transcriptional states, indicating that canonical mutations alone cannot fully account for the diversity of clinical outcomes (10).

RNA 5-methylcytosine (m^5^C) provides a dynamic regulatory layer, distinct from DNA sequence alterations, through which such cellular plasticity may be understood. Transcriptome-wide m^5^C deposition, together with its installation and recognition, can influence RNA abundance, nuclear export, stability and translation (11–13). Y-box-binding protein 1 (*YBX1*) has been established as an m^5^C reader that recognises modified transcripts and protects them from degradation in diverse biological contexts (14–16). Its broad RNA-binding capacity, together with evidence that it cooperates with NSUN-family methyltransferases to stabilise cancer-related mRNAs, positions *YBX1* as a plausible molecular link between RNA modification and transcript fate (17–19). *NSUN5* is best known for catalysing m^5^C modification of ribosomal RNA and thereby regulating protein synthesis and adaptation to cellular stress (20, 21). More recently, *NSUN5* has also been implicated in tumour immune evasion and in the progression of renal and liver cancers (22–24). Direct evidence further supports its ability to regulate cancer-associated mRNAs through m^5^C modification (25). Centromere protein F (*CENPF*), in turn, functions as a downstream effector of cell-cycle progression and chromosome segregation and can cooperate with *FOXM1*-associated regulatory networks to sustain malignant proliferation (26, 27). Its prognostic association and transcriptional regulation in cutaneous melanoma further support *CENPF* as a candidate downstream node (28, 29).

Paired sequencing of tumour and normal tissues from the same patient can capture coordinated changes in RNA modification and gene expression. However, tissue-averaged signals alone cannot determine which cell populations harbour these alterations, which malignant states they represent, or whether they are linked to the surrounding immune and stromal ecosystem. Single-cell atlases of melanoma have revealed substantial complexity in malignant cell states, T-cell exclusion and treatment-persistent transcriptional programmes (30–32). Studies of transcriptional regulation and cellular trajectories have further identified continuous relationships amongst invasive state transitions, recurrent melanoma cell states and dedifferentiation from benign to malignant phenotypes (33–35). Immune-cell composition in treatment cohorts is likewise closely associated with responses to immune checkpoint blockade (36). Integrating bulk cohorts, single-cell and spatial transcriptomic data, and gene-regulatory networks is therefore necessary to localise epitranscriptomic alterations to malignant cell populations, define their tissue context and establish priorities for state-directed therapeutic intervention.

In this study, we first jointly analysed m^5^C methylated RNA immunoprecipitation sequencing (m^5^C-MeRIP-seq) and RNA sequencing data from patient-matched melanoma and normal tissues. On the basis of coordinated changes in CENPF m^5^C enrichment and expression, we proposed a NSUN5–YBX1–CENPF (NYC) working programme. We then experimentally anchored the key molecular associations in patient tissues and cellular models and integrated TCGA, single-cell and spatial transcriptomic datasets to characterise the clinical phenotype, cellular origin, regulatory architecture and tumour ecosystem of the NYC programme. Finally, we combined bulk transcriptomic signature reversal with single-cell drug-connectivity analysis in a two-stage screening framework to prioritise therapeutic candidates predicted to selectively reverse the NYC-High state.

## 2 Results

### 2.1 Integrated analysis of paired transcriptomic and epitranscriptomic profiles identifies a candidate NSUN5–YBX1–CENPF programme

To derive experimentally testable molecular hypotheses from our in-house sequencing data, we performed parallel m^5^C-MeRIP-seq and RNA sequencing on three patient-matched pairs of melanoma and adjacent normal tissues. Candidate molecular relationships were jointly prioritised on the basis of three criteria: increased m^5^C enrichment, altered transcript abundance and covariation with potential m^5^C recognition or regulatory factors (**Figure 1**). Within this discovery framework, *CENPF* showed higher peak-call counts and transcript abundance in melanoma, motivating a paired analysis of regional enrichment relative to local Input abundance. The m^5^C methyltransferase *NSUN5* and *YBX1*, considered here as a candidate reader, provided a plausible upstream regulatory framework for these changes. On the basis of these observations, we defined *NSUN5*, *YBX1* and *CENPF* as the NYC three-gene programme and subsequently evaluated its biological and translational relevance in melanoma using clinical cohorts, tissue- and cell-based experiments, and multiscale bioinformatic analyses.

**Figure 1:**
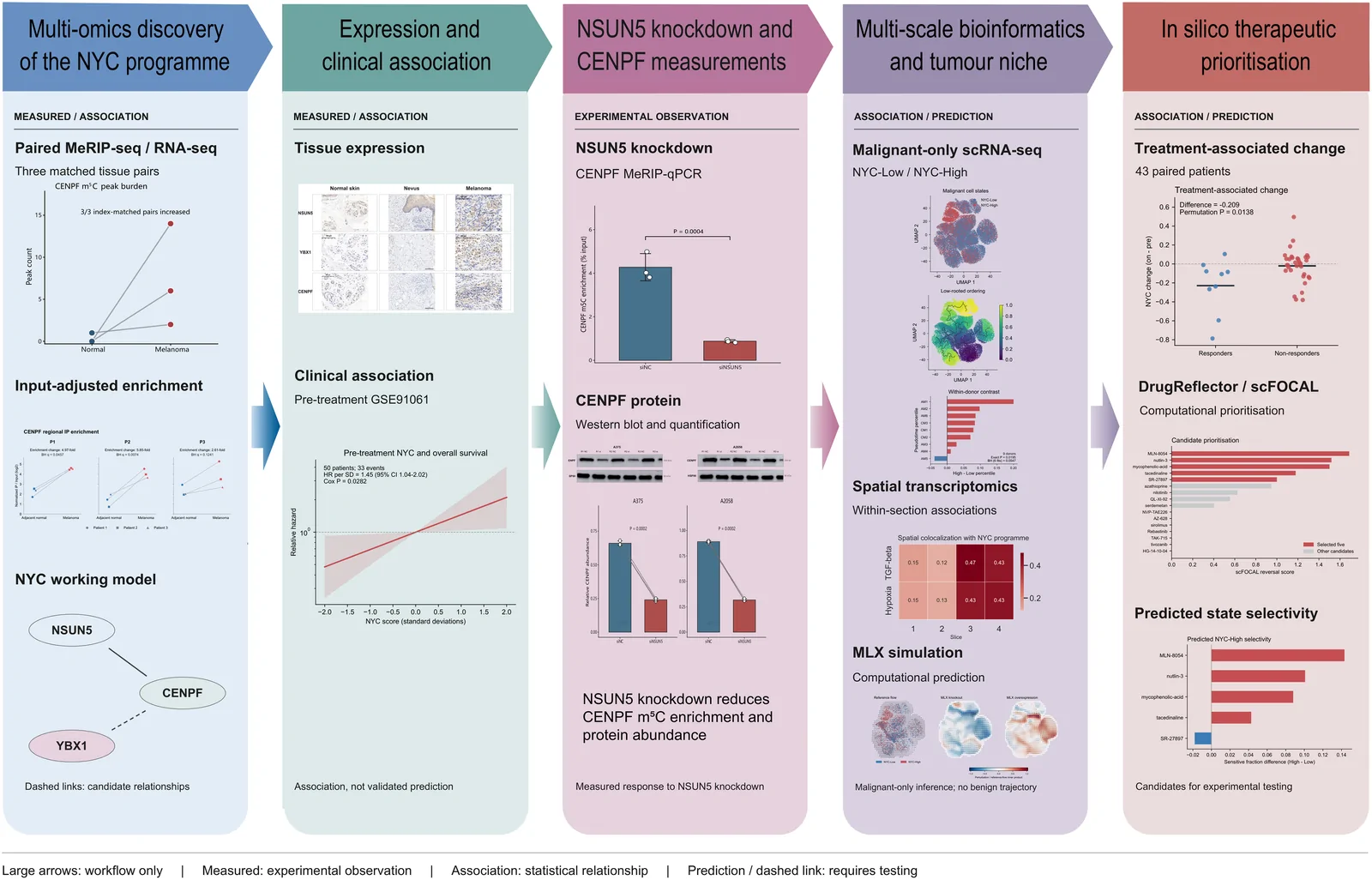
Study workflow and evidence levels of the NYC programme. The five modules distinguish measured observations, statistical associations and computational predictions. Module 1 summarises paired MeRIP-seq and Input RNA measurements from three melanoma-adjacent-normal tissue pairs, Input-adjusted regional enrichment and the candidate NYC working model. Dashed links denote candidate molecular relationships; the YBX1-CENPF interaction has not been established experimentally. Module 2 presents tissue expression and the pretreatment GSE91061 survival association; the displayed 50-patient analysis uses the revision-stage one-month survival restriction described in Methods and does not establish validated clinical prediction. Module 3 shows measured changes in CENPF MeRIP-qPCR enrichment and protein abundance after NSUN5 knockdown. Module 4 presents malignant-only NYC-Low/NYC-High state analyses, exploratory Low-rooted ordering, within-section spatial associations and MLX perturbation simulations; these do not demonstrate benign-to-malignant development or causal regulation. Module 5 separates treatment-associated NYC change in 43 paired patients from computational DrugReflector/scFOCAL prioritisation and predicted state selectivity. Large arrows indicate workflow sequence only, not causal relationships or increasing levels of evidence. Quantitative details are reported in the corresponding main figures and Methods.

The subsequent analyses were structured around three interrelated questions. First, is m^5^C enrichment of *CENPF* mRNA reproducibly detected in melanoma, and is it associated with expression of the three NYC genes and patient outcomes? Second, does high NYC activity define a distinct malignant cell state with a characteristic tumour microenvironment and regulatory architecture? Third, can the transcriptional features of the NYC-High state be used to prioritise candidate compounds with the potential to reverse this state? This framework established a continuous line of investigation extending from discovery in patient-matched sequencing data and experimental validation to single-cell and spatial dissection, followed by in silico perturbation and drug prioritisation.

### 2.2 Paired Input-adjusted analysis identifies increased CENPF regional IP enrichment

Across three melanoma specimens (A1–A3) and their matched adjacent normal tissues (B1–B3), the number of m^5^C peaks assigned to *CENPF* was markedly higher in the tumour samples. Specifically, 14, 6 and 2 *CENPF*-associated peaks were detected in A1, A2 and A3, respectively, whereas 0, 0 and 1 peak were detected in B1, B2 and B3, respectively. In aggregate, 22 peaks were identified in the melanoma tissues, compared with only one in the matched normal tissues (**Figure 2A**). Sample-resolved interval tracks showed the positions of called peaks across the *CENPF* locus (**Figure 2B**). These tracks display peak calls rather than read-coverage intensity.

**Figure 2:**
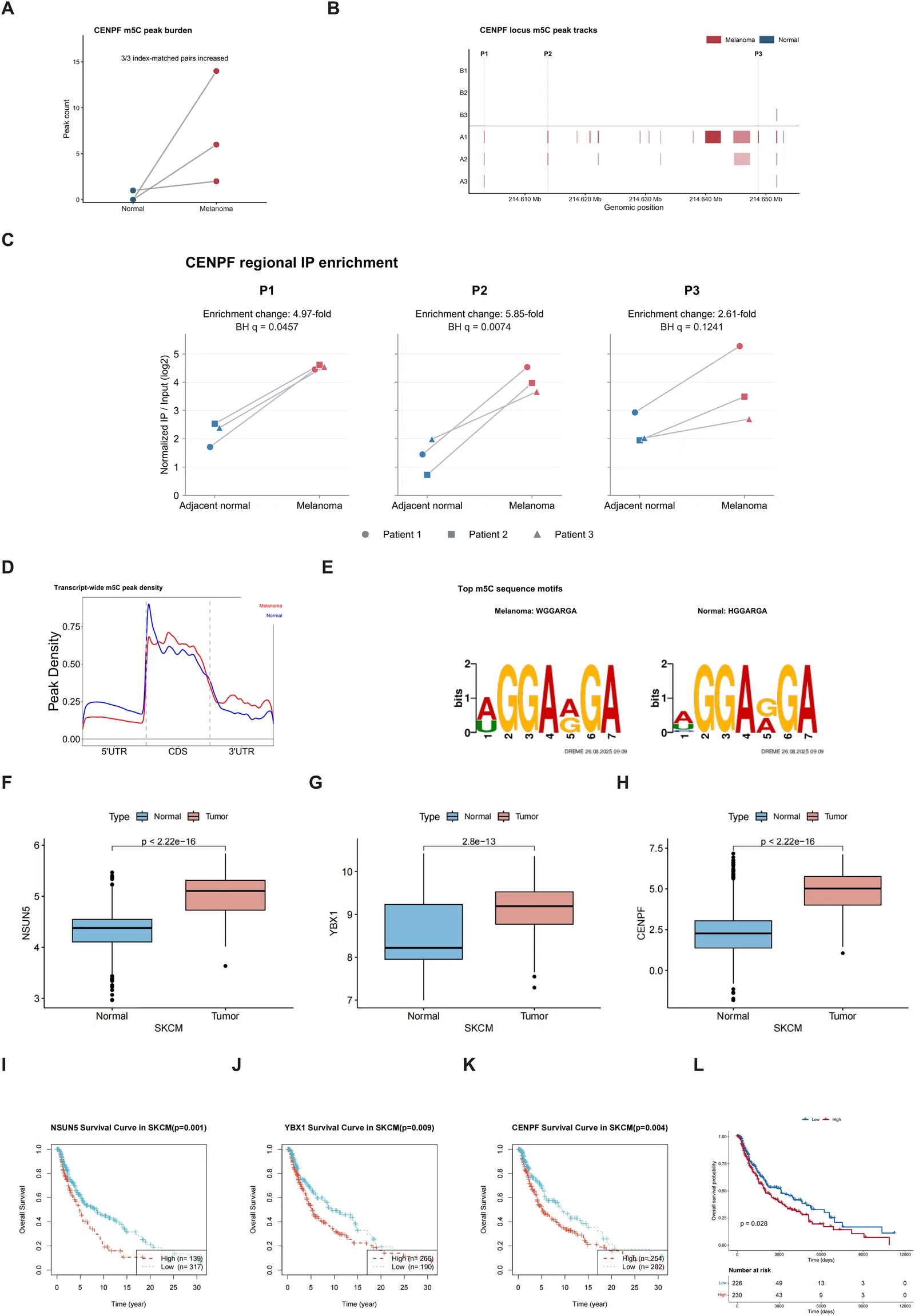
Paired CENPF enrichment reanalysis and clinical associations of the NYC programme **(A)**, Number of CENPF-associated m^5^C peaks called in three patient-matched pairs of adjacent normal tissues **(B1–B3)** and melanoma tissues **(A1–A3)**. Lines connect matched samples. **(B)**, Positions of sample-resolved m^5^C peak calls across the CENPF locus; these are called intervals rather than read-coverage tracks. **(C)**, Background-normalised IP/Input ratios at P1–P3 in three matched patient pairs. Shapes identify patients and lines join matched tissues. Enrichment fold changes and BH q values derive from the paired edgeR IP-by-condition interaction model, with correction across 7,937 candidate regions; they are not tests performed directly on the displayed ratios. **(D)**, Transcriptome-wide metagene density of m^5^C peaks across the 5′ UTR, CDS and 3′ UTR in normal and melanoma samples. **(E)**, Top-ranked DREME motifs identified in the two tissue groups. **(F–H)**, Differential expression of NSUN5, YBX1 and CENPF between normal skin and SKCM tissues. **(I–K)**, Overall-survival curves stratified by high versus low expression of NSUN5, YBX1 and CENPF. **(L)**, Overall survival in the TCGA-SKCM cohort stratified at the median NYC ssGSEA score; n = 456, HR = 1.348, 95% CI 1.032–1.760, P = 0.0283.

The three originally nominated CENPF regions were P1 (chr1:214603194–214603321), P2 (chr1:214613713–214613916) and P3 (chr1:214648674–214648827), using zero-based, half-open BED coordinates. Paired edgeR quasi-likelihood interaction analysis estimated melanoma-to-normal relative IP/Input enrichment changes of 4.97, 5.85 and 2.61, respectively (**Figure 2C**). P1 and P2 met the BH threshold across 7,937 candidate regions (q = 0.0457 and 0.00742), whereas P3 did not (q = 0.1241). Patient-resolved normalised IP/Input ratios are shown alongside the model estimates. P1 was called in all three melanoma samples, P2 in two, and P3 predominantly in A1 (**Supplementary Figures S1A, B**). These results support regional relative enrichment changes after accounting for local Input abundance; they do not measure absolute methylation fractions. Full edgeR results and counts are supplied in Supplementary Data R2.

Transcriptome-wide metagene analysis revealed distinct distributions of m^5^C peak density across the 5′ UTR, CDS and 3′ UTR between melanoma and normal tissues (**Figure 2D**). DREME analysis identified WGGARGA and HGGARGA as the top-ranked enriched sequence motifs across the two groups (**Figure 2E**), delineating the sequence context of m^5^C-enriched regions in the respective tissue types. Paired analysis of the Input libraries estimated a 2.20-fold increase in *CENPF* transcript abundance (*P* = 0.00313; genome-wide BH FDR = 0.132). Across all six sequenced specimens, *YBX1* expression correlated positively with *CENPF* expression (Spearman’s ρ = 0.829, *P* = 0.0416). These expression and regional-enrichment observations provide discovery-level support for the NYC working model; the six-specimen expression correlation remains exploratory.

### 2.3 Elevated expression and composite activity of the NYC programme are associated with adverse outcomes in melanoma

Publicly available expression datasets further supported aberrant activation of the three NYC genes in melanoma. Compared with normal skin, *NSUN5*, *YBX1* and *CENPF* were all expressed at significantly higher levels in skin cutaneous melanoma tissues (*NSUN5*, *P* < 2.22 × 10^−16^; *YBX1*, *P* = 2.8 × 10^−13^; *CENPF*, *P* < 2.22 × 10^−16^; **Figures 2F–H**). Kaplan–Meier analyses using gene-specific optimised cut-offs showed that high expression of *NSUN5*, *YBX1* and *CENPF* was individually associated with poorer overall survival (*P* = 0.001, 0.009 and 0.004, respectively; **Figures 2I–K**). Cross-cohort Cox analyses showed a broadly concordant direction of association with adverse prognosis, although the magnitude of the effects varied amongst patient populations (**Supplementary Figures S1C–E**).

To assess the clinical relevance of the three genes as a unified biological programme, we calculated an NYC ssGSEA score using the TCGA-SKCM log-transformed TPM expression matrix, which comprised 19,344 genes and 473 patients. Patients were subsequently dichotomised at the median score into NYC-High (n = 236) and NYC-Low (n = 237) groups. Amongst the 456 patients with complete survival information, the NYC-High group had significantly shorter overall survival than the NYC-Low group (Cox HR = 1.348, 95% CI 1.032–1.760, P = 0.0283; **Figure 2L**). Schoenfeld residual testing showed no evidence of violation of the proportional-hazards assumption, with P = 0.91 for both the group-specific and global tests. These results indicate that coordinated high activity of the NYC programme delineates a subgroup of patients with melanoma who have an unfavourable clinical outcome. These unadjusted associations were reassessed in a common complete-case set of 378 patients with 178 deaths. NYC correlated with Hallmark G2M checkpoint and E2F target scores (Spearman ρ = 0.734 and 0.747). The clinical-covariate-adjusted HR per SD of NYC was 1.22 (95% CI 1.04–1.43; nominal P = 0.0136), but the association did not persist after G2M or E2F adjustment (P = 0.478 and 0.580). The data therefore do not establish proliferation-independent prognostic value (Supplementary Data R1).

### 2.4 Tissue- and cell-based experiments support *NSUN5*-dependent m^5^C enrichment of *CENPF* and maintenance of *CENPF* protein abundance

We first assessed the expression of the three NYC genes at the tissue and cellular levels. Representative high-magnification fields, each traceable to its source whole-slide image, showed an overall increase in immunohistochemical staining for *NSUN5*, *YBX1* and *CENPF* from normal skin through benign naevi to melanoma, with more extensive positive staining in melanoma regions (**Figure 3A**). Corresponding whole-slide overviews are provided in **Supplementary Figure S2**. These histological patterns were consistent with the direction of expression changes observed in public transcriptomic datasets.

**Figure 3:**
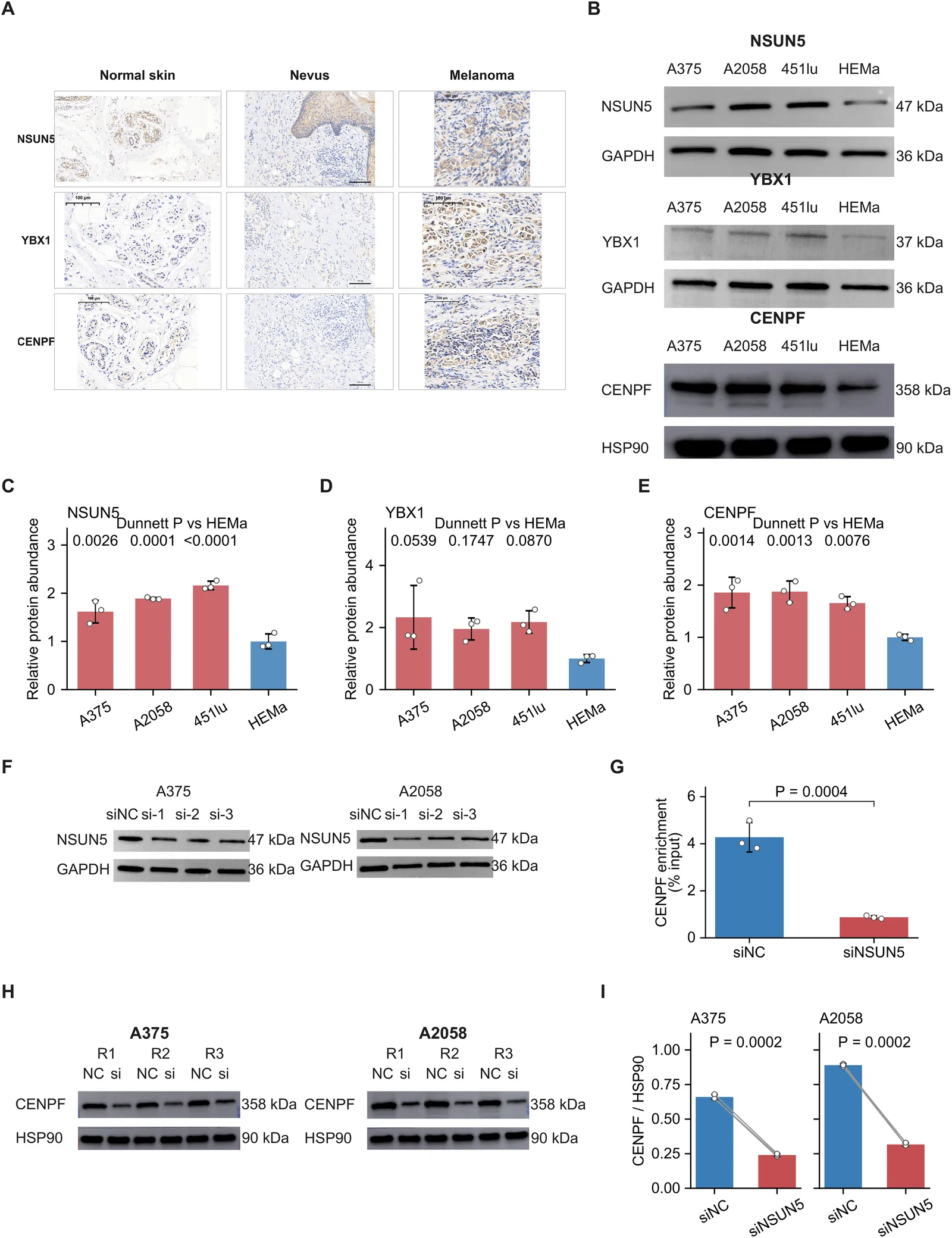
Tissue- and cell-based experiments support NSUN5-dependent m^5^C enrichment of CENPF and CENPF protein abundance. **(A)**, Representative high-magnification immunohistochemical images of NSUN5, YBX1 and CENPF in normal skin, benign naevi and melanoma. All displayed fields were traced to their corresponding source whole-slide images. **(B)**, Baseline western blots of HEMa, A375, A2058 and 451Lu cells. **(C–E)**, Densitometric quantification of baseline NSUN5, YBX1 and CENPF protein abundance. Data are shown as the mean ± s.d. with individual values from three independent biological replicates. Statistical significance was assessed by one-way ANOVA followed by Dunnett’s multiple-comparison test, with HEMa serving as the common control. **(F)**, Western blot screening of three independent siRNAs targeting NSUN5 in A375 and A2058 cells. **(G)**, CENPF-targeted MeRIP–qPCR following NSUN5 knockdown. Displayed values are % input; n = 3; statistical testing used log_2_(% input) values with a two-sided Welch’s t-test, P = 0.0004. **(H)**, Western blot analysis of CENPF following NSUN5 knockdown in A375 and A2058 cells. **(I)**, Paired densitometric quantification of the blots shown in H; n = 3; two-sided paired t-test, P = 0.0002 for each cell line. The displayed WB crops retain the terminal lanes in full. For H, R1-R3 identify the three paired experiments; NC and si denote siNC and siNSUN5, respectively. Source-image frames are available in **Supplementary Figures 3–5** and the separate WB source archive.

Baseline western blotting was subsequently performed in HEMa melanocytes and the A375, A2058 and 451Lu melanoma cell lines. *NSUN5* and *CENPF* protein abundance was higher in all three melanoma cell lines than in HEMa cells (**Figures 3B–E**). Based on three independent biological replicates, one-way analysis of variance followed by Dunnett’s multiple-comparison test yielded *P* values of 0.0026, <0.0001 and <0.0001 for *NSUN5* in A375, A2058 and 451Lu cells, respectively. The corresponding *P* values for *CENPF* were 0.0014, 0.0013 and 0.0076. *YBX1* protein abundance also showed an upward trend in all three melanoma cell lines, although the adjusted *P* values did not reach statistical significance (A375, *P* = 0.0539; A2058, *P* = 0.1747; 451Lu, *P* = 0.0870). Full source blots and results from individual replicates are presented in **Supplementary Figure S3**.

We next screened three independent siRNAs targeting *NSUN5* in A375 and A2058 cells. All three siNSUN5 constructs reduced *NSUN5* protein abundance, with decreases of approximately 12.1–32.0% in A375 cells and 43.4–51.4% in A2058 cells (**Figure 3F**; **Supplementary Figure S4**), thereby establishing directionally consistent knockdown conditions for subsequent experiments. Following *NSUN5* knockdown, m^5^C immunoprecipitation enrichment of *CENPF* mRNA was significantly reduced, as measured by MeRIP–qPCR (*n* = 3; two-sided Welch’s *t*-test, *P* = 0.0004; **Figure 3G**). This finding supports an *NSUN5*-dependent component of *CENPF* mRNA m^5^C enrichment. Consistent with this result, *CENPF* protein abundance decreased significantly after *NSUN5* knockdown in both A375 and A2058 cells (paired *n* = 3; two-sided paired *t*-test, *P* = 0.0002 for each cell line; **Figures 3H, I**). Amplification and melting curves, together with the source blots for *CENPF*, are provided in **Supplementary Figure S5**. Concordant changes in *YBX1* following siNSUN5 treatment across two independent experiments are shown in **Supplementary Figure S6**.

Taken together, the in-house tissue observations, baseline protein-expression data, MeRIP–qPCR results and post-knockdown protein measurements provide complementary evidence that *NSUN5* contributes to the maintenance of *CENPF* mRNA m^5^C enrichment and *CENPF* protein abundance. These findings further position *YBX1* as a candidate m^5^C-recognition and transcript-stabilising factor that covaries with this programme, although its direct functional involvement remains to be established.

### 2.5 NYC-High melanomas exhibit an immune-hyporesponsive tumour ecosystem

Using the same NYC stratification in TCGA-SKCM, we systematically compared tumour-microenvironment cell-type signatures, immune-exclusion and immunosuppression programmes, and biomarkers associated with response to immunotherapy between the NYC-High and NYC-Low groups (**Figures 4A–D**). NYC-High tumours showed broadly reduced signatures of T-cell infiltration and activation, including CD4^+^ memory and effector states and CD8^+^ effector activity, together with lower signatures of natural killer cells, dendritic cells and selected myeloid populations (**Figure 4A**). Immune-exclusion and immunosuppression modules did not reveal a uniform increase in any single cell population or pathway. Instead, they indicated a broader reorganisation of fibroblast/TGF-β-associated, myeloid and regulatory immune features (**Figures 4B, C**). Consistent with these observations, scores reflecting inflammatory activity or the potential for response to immunotherapy—including the T-cell-inflamed gene-expression profile, CD8 T-effector signature, immune-checkpoint signature, immunophenoscore and Pan-F-TBRs—were generally lower in the NYC-High group, whereas mismatch-repair-related features were relatively increased (**Figure 4D**).

**Figure 4:**
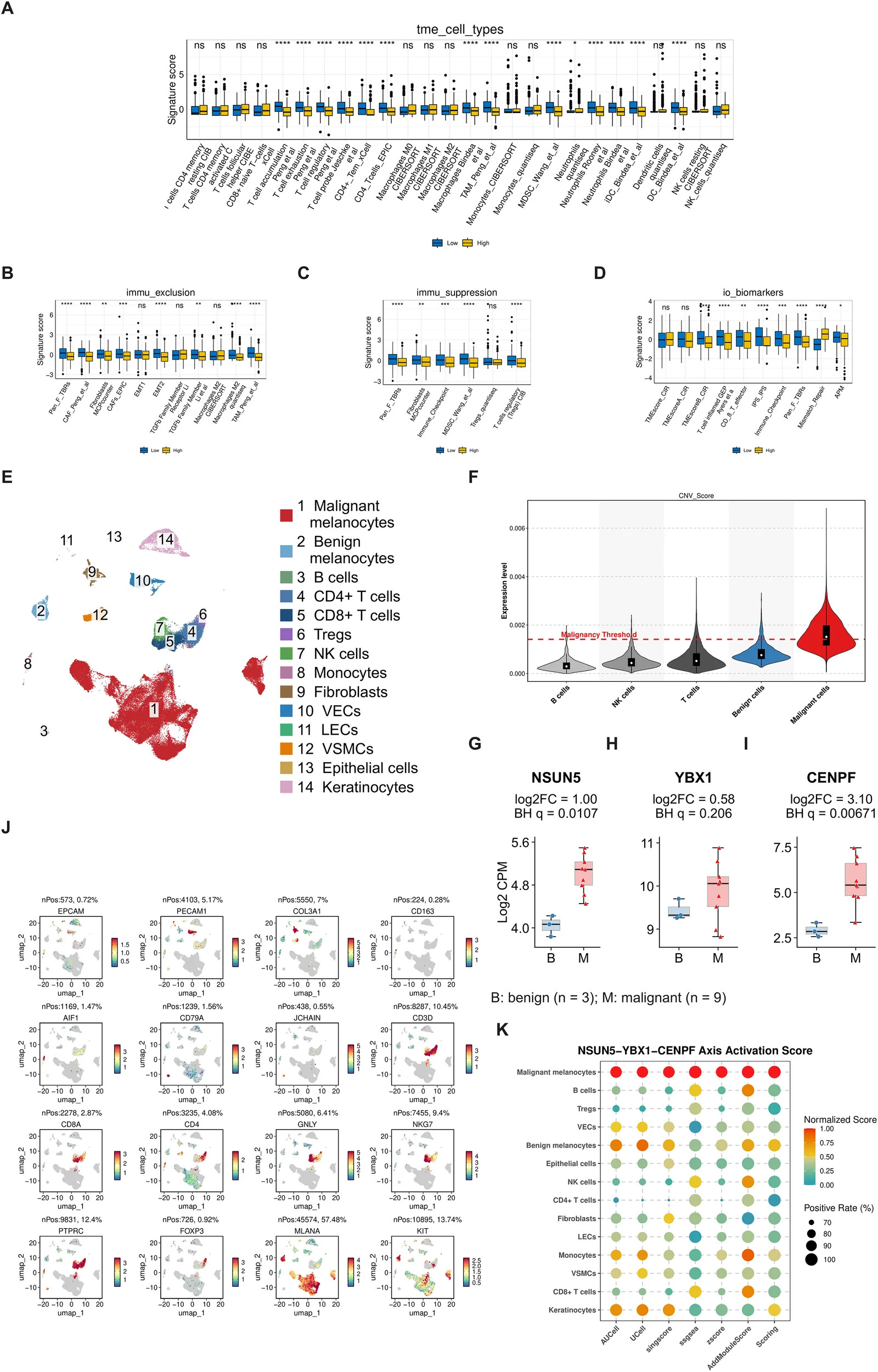
Immune-hyporesponsive ecology of NYC-High melanoma and single-cell localisation of the NYC programme. **(A)**, Tumour-microenvironment cell-type signatures in NYC-Low and NYC-High tumours from TCGA-SKCM. **(B, C)**, Immune-exclusion- and immunosuppression-related signatures in the two NYC groups. **(D)**, Biomarker scores associated with immunotherapy response. For A–D, boxes indicate the interquartile range, centre lines indicate the median, and whiskers extend to 1.5 times the interquartile range. Comparisons were performed using two-sided Wilcoxon rank-sum tests, followed by Benjamini–Hochberg correction within the corresponding functional module. ns, adjusted P ≥ 0.05; *P < 0.05; **P < 0.01; ***P < 0.001; ****P < 0.0001. **(E)**, UMAP representation of the final cell-type annotations after integration of benign-naevus and melanoma samples. **(F)**, inferCNV-derived CNV scores across the major cell populations. The red dashed line indicates the prespecified threshold for malignant classification. **(G–I)**, Raw-UMI pseudobulk expression of NSUN5, YBX1 and CENPF in three benign-naevus specimens (B, blue) and nine independent melanoma patients (M, red; one specimen per patient). Each point is a specimen, not a cell. The repeated AM3_post specimen was excluded from this independent-patient contrast; AM3_pre was retained. Boxes show the interquartile range, centre lines the median, and whiskers 1.5 times the interquartile range. The y axis is TMM log2 CPM. Annotations report edgeR quasi-likelihood log2 fold changes (malignant versus benign) and BH q values across NSUN5, YBX1 and CENPF. YBX1 is not significantly different. Genome-wide FDR and complete sample-level results are in Supplementary Data R4. **(J)**, Feature plots of representative marker genes used for final cell-type annotation. **(K)**, NYC programme activity calculated using AUCell, UCell, singscore, ssGSEA, z-score-based scoring and AddModuleScore. The integrated NYC score represents the mean score across the six methods. Colour indicates the normalised score within each cell type, and circle size denotes the proportion of score-positive cells. Benign versus malignant comparisons in G–I are confounded by dataset origin and are not treated as independent disease-effect tests. InferCNV references are immune populations, not the benign-naevus cells. The atlas in E uses the unchanged original Harmony-derived coordinates and all 79,133 cells; only display formatting was revised.

Thus, the predominant immune phenotype of NYC-High melanoma was not characterised by the expansion of a single immunosuppressive cell population, but rather by coordinated attenuation across multiple axes related to effector immunity, inflammation and therapeutic responsiveness. These bulk-level findings link high NYC activity to an immune-hyporesponsive tumour ecosystem and provide a basis for tracing the cellular origin of this signal at single-cell resolution. These are unadjusted bulk associations, not evidence of an independent response biomarker. Additional proliferation- and tumour-purity-adjusted analyses are provided in Supplementary Data R1; robust independence was not established for the bulk immune associations.

### 2.6 Single-cell mapping localises high NYC activity to malignant melanocytic cells

We integrated three benign-naevus samples from GSE277165 with ten melanoma samples from GSE215120. Following ambient RNA correction, quality control, doublet removal, batch integration, clustering and marker-based annotation, 79,133 high-quality cells were retained, comprising 12,876 cells from benign samples and 66,257 cells from melanoma samples. These cells were assigned to 14 major cell populations (**Figure 4E**). Using immune cells as the reference population, inferCNV analysis showed that the candidate malignant melanocytic cells had the highest CNV scores, which were generally above the prespecified threshold for malignant classification, supporting their malignant identity (**Figure 4F**).

Within the melanocytic compartment, we replaced cell-level comparisons with raw-count pseudobulk summaries from three benign-naevus specimens and nine independent melanoma patients (one specimen per patient; **Figures 4G–I**). NYC-Low and NYC-High cells were pooled within each malignant specimen. NSUN5 and CENPF had higher pseudobulk expression in the malignant group (log2 fold changes 1.00 and 3.10; BH q = 0.0107 and 0.00671 across the three target genes), whereas YBX1 did not show a significant difference (log2 fold change 0.58; q = 0.206). These sample-level results avoid treating cells as independent biological replicates, but disease status remains confounded with study of origin. They are exploratory cross-study summaries, not disease-specific differential-expression validation or evidence of a benign-to-malignant trajectory. Complete results and within-GSE277165 sensitivity analyses are provided in Supplementary Data R4. Feature plots of canonical markers supported the retained melanocytic, immune, endothelial and stromal annotations (**Figure 4J**).

To minimise dependence on any single scoring method, we quantified NYC programme activity using six algorithms: AUCell, UCell, singscore, ssGSEA, z-score-based scoring and AddModuleScore. The mean of the six resulting scores was defined as the integrated NYC score. All six individual methods, as well as the integrated score, consistently identified malignant melanocytic cells as the population with the highest normalised NYC activity and the largest proportion of NYC-positive cells, whereas immune and stromal populations generally exhibited low activity (**Figure 4K**).

Amongst 46,194 malignant melanocytic cells, the global mean of the integrated NYC score was used as the threshold to define NYC-High (n = 17,356) and NYC-Low (n = 28,838) states. This stratification defined the malignant-cell expression states of the NYC programme and provided a unified state definition for subsequent analyses of cell–cell communication, metabolism, cellular trajectories, regulatory networks and predicted drug connectivity.

Within the original malignant cohort, patient-aware models related NYC state to an author-defined immune-resistance programme (31) after adjustment for G2M, E2F and transcript depth. The mean standardised NYC-High effect was 0.131 (95% CI 0.065–0.198; nine of nine patients positive; exact sign-flip P = 0.00391; BH q = 0.0156 across the 12-endpoint comparison). Removing proliferation-associated genes preserved the direction and statistical support (effect 0.119, 95% CI 0.051–0.188; q = 0.0156). These findings concern a malignant-cell transcriptional association rather than a demonstrated clinical resistance mechanism (Supplementary Data R1).

### 2.7 Inferred MIF, LAMININ and CD99 communication networks link the NYC state to immune–stromal remodelling

Using the single-cell dataset restricted to melanoma samples, we first inferred all CellChat signalling pathways without *a priori* selection. We then prioritised the MIF, LAMININ and CD99 communication axes for detailed presentation on the basis of their differences between NYC-High and NYC-Low states and their biological relevance. The inferred MIF network connected NYC-High and NYC-Low malignant melanocytic cells with both myeloid and lymphoid populations, suggesting that variation in tumour-cell state is coupled to immunoregulatory signalling. The LAMININ network was dominated by interactions amongst malignant cells, fibroblasts and vascular-associated populations, consistent with remodelling of the basement membrane and extracellular matrix. By contrast, CD99 formed an extensive cell-contact signalling network spanning malignant, immune and stromal compartments (**Figures 5A–I**). Circular network plots, signalling-role heatmaps and ligand–receptor chord diagrams characterised these patterns at the levels of overall interaction strength, sender and receiver roles, and individual molecular pairs, respectively.

**Figure 5:**
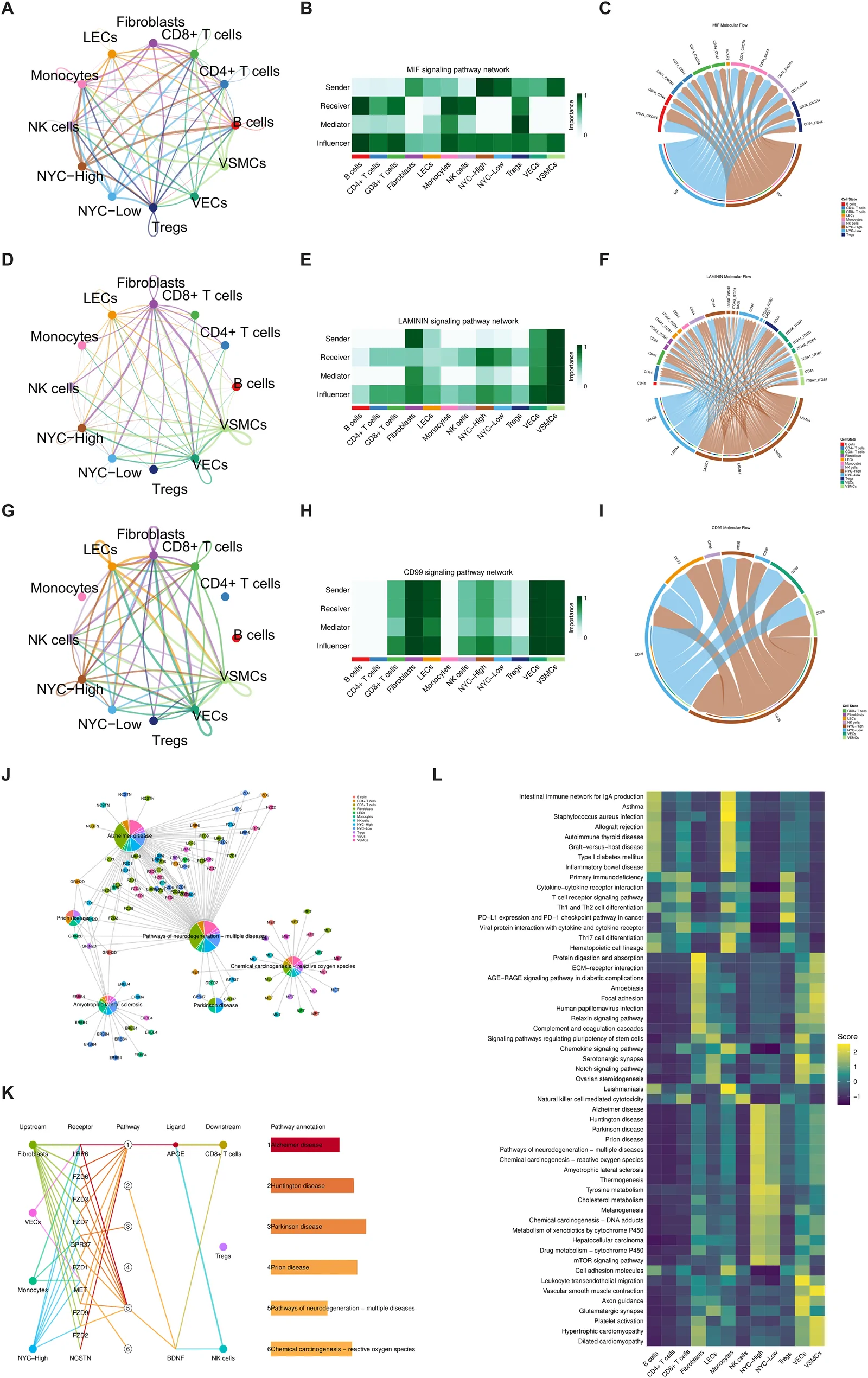
NYC-state-associated intercellular communication and intracellular pathway networks. **(A–C)**, Circular communication network, signalling-role heatmap and ligand–receptor chord diagram for the MIF pathway. **(D–F)**, Corresponding analyses for the LAMININ pathway. **(G–I)**, Corresponding analyses for the CD99 pathway. **(J)**, CommPath PathNet analysis with NYC-High malignant cells specified as the receiver state. Major receptor nodes include *MET*, LRP6, members of the FZD family, *GPR37*, *ERBB4*, *GRIN2D* and *NCSTN*. Coloured sectors within circular pathway nodes indicate the contributions of different sender cell populations. **(K)**, Communication chains originating from fibroblasts, VECs, monocytes and NYC-High malignant cells, passing through the indicated receptors and six KEGG-annotated pathway modules, and subsequently linking to CD8^+^ T cells and NK cells through *APOE* or *BDNF*. **(L)**, Normalised pathway-activity heatmap across 12 cell populations. Yellow indicates relatively high activity, whereas purple indicates relatively low activity.

CommPath further linked extracellular communication signals to intracellular pathway responses in receiver cells. In the PathNet analysis with NYC-High cells specified as the receiver state, inputs from malignant cells and multiple microenvironmental populations converged primarily on receptor nodes including *MET*, LRP6, *FZD1*, *FZD2*, *FZD3*, *FZD6*, *FZD7*, *FZD9*, *GPR37*, *ERBB4*, *GRIN2D* and *NCSTN*. These receptor-associated signals were linked to KEGG modules annotated as Chemical carcinogenesis—reactive oxygen species, Alzheimer disease, Parkinson disease, Prion disease, Amyotrophic lateral sclerosis and Pathways of neurodegeneration—multiple diseases (**Figure 5J**). In this context, these disease-labelled annotations represent shared gene modules related to oxidative stress, mitochondrial biology and proteostasis; they should not be interpreted as evidence that melanoma exhibits the corresponding neurological disorders.

A chained network centred on the NYC-High state further delineated continuous communication routes in which fibroblasts, vascular endothelial cells (VECs), monocytes and NYC-High malignant cells acted as upstream sources, signals were transmitted through the above receptor nodes and intracellular pathway modules, and *APOE* or *BDNF* provided downstream links to CD8^+^ T cells and natural killer cells. Although regulatory T cells were included as candidate downstream populations, they did not form a major retained communication chain under the applied filtering thresholds (**Figure 5K**).

The global pathway-activity heatmap revealed marked cell-type specialisation. Immune-response modules were concentrated predominantly in monocytes, CD8^+^ T cells and regulatory T cells, whereas ECM–receptor interaction and focal adhesion were more active in fibroblasts and vascular smooth muscle cells (VSMCs). Leukocyte transendothelial migration and vascular smooth muscle contraction were preferentially associated with VECs and VSMCs, respectively. Compared with NYC-Low cells, NYC-High malignant cells showed higher relative activity of reactive oxygen species-related, mTOR and several metabolic modules (**Figure 5L**). Thus, NYC-High represents not only a tumour-cell-intrinsic transcriptional state but also a communication node that receives stromal, vascular and myeloid inputs and interfaces with cytotoxic lymphocyte populations. Its intracellular stress and metabolic programmes were concordant with an immune-hyporesponsive and tissue-remodelling ecosystem.

### 2.8 Spatial co-occurrence and predicted metabolic fluxes delineate TGF-β, hypoxic and metabolic features of regions with high NYC activity

To examine the tissue-level spatial context of the NYC programme, we analysed three primary melanoma sections and one melanoma brain-metastasis section. NYC, Hallmark TGF-β signalling and Hallmark hypoxia scores each displayed pronounced within-section spatial heterogeneity and overlapped in multiple tumour regions (**Figure 6A**). Section-wise Spearman correlation analyses yielded correlation coefficients of 0.155, 0.123, 0.475 and 0.425 between NYC and TGF-β scores, and 0.148, 0.131, 0.425 and 0.430 between NYC and hypoxia scores, respectively (**Figure 6B**). The first two sections showed weak positive correlations, whereas the latter two showed moderate positive correlations. These findings suggest that regions with high NYC activity tend to coincide with TGF-β- and hypoxia-related transcriptional features across specimens, although the strength of these associations varies spatially and between cases.

**Figure 6:**
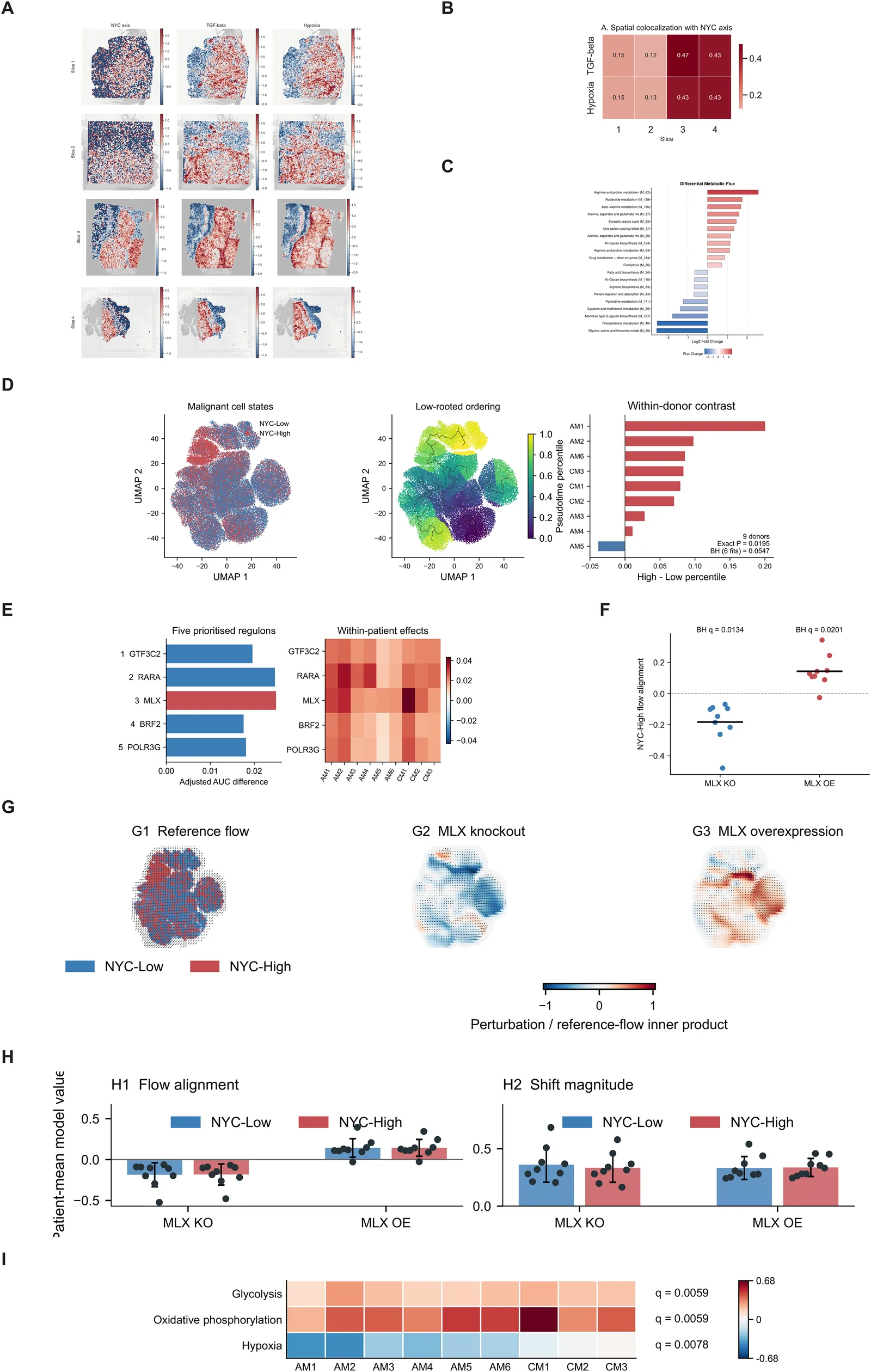
Spatial context and malignant-cell regulatory analyses of the NYC programme. **(A)**, Spatial distributions of NYC, TGF-beta and hypoxia scores across four melanoma sections. **(B)**, Within-section Spearman correlations. **(C)**, scFEA-predicted metabolic modules in NYC-High versus NYC-Low malignant cells. **(D)**, Malignant-only M05 embedding and exploratory Low-rooted principal graph, with patient-level differences in median pseudotime percentile; all 46,194 malignant cells from ten specimens and nine patients were retained. The two AM3 specimens were combined at the patient level. P is the exact paired signed-rank value; the displayed BH value concerns six configurations, not the complete historical parameter search. **(E)**, Five highest-ranked eligible regulons and S-phase/G2M-adjusted activity differences across patients. **(F)**, Simulated MLX knockout (KO) and overexpression (OE) flow alignment in NYC-High cells; points represent nine patient summaries and black lines their means. BH correction covers 72 signed endpoints. G1, Reference pseudotime flow over observed NYC-Low (blue) and NYC-High (red) cells. G2-G3, Simulated MLX KO and OE vector fields over grid-level perturbation/reference-flow inner products. A shared, zero-centred colour scale indicates opposition (blue, negative) or alignment (red, positive) with reference flow, not NYC state or statistical significance. KO/OE share an arrow scale; the reference field uses its own scale. H1-H2, Patient-level flow alignment and shift magnitude after simulated MLX KO/OE. Blue bars indicate NYC-Low and red bars NYC-High. Each point represents one patient (n = 9 per state); bars and error bars show mean and standard deviation. Alignment retains direction relative to reference flow; magnitude measures displacement size without direction. **(I)**, Patient-level metabolic associations with MLX regulon activity, adjusted for NYC, G2M, E2F and depth, with overlapping target genes excluded. Heatmap entries are individual patient effects; annotations give BH q values across the three metabolic endpoints. Panels **(D–I)** exclude benign melanocytes and do not establish causal development, regulation or metabolic mediation. Panel **(I)** displays the patient-specific adjusted association heatmap and the three-endpoint BH q values. Complete numerical effects and confidence intervals remain in Supplementary Data R1. Display resizing does not change the stored perturbation vectors or their underlying statistics.

Within malignant melanocytic cells, scFEA further predicted directional differences in metabolic flux across multiple modules between the NYC-High and NYC-Low states (**Figure 6C**). The affected metabolic programmes implicated hypoxic adaptation, energy utilisation and biosynthetic demand, providing additional functional context for the proliferative capacity and microenvironmental adaptation of NYC-High cells. Together, the spatial co-occurrence of functional programmes and single-cell metabolic-flux predictions indicate that high NYC activity is associated with a recognisable tissue ecosystem and metabolic reprogramming.

### 2.9 Malignant-cell trajectory and donor-aware regulatory analyses prioritise candidate upstream regulators

We restricted trajectory and downstream regulatory inference to the 46,194 malignant melanocytic cells in GSE215120, retaining all malignant cells from 10 specimens representing nine donors. The frozen M05 embedding yielded a connected principal graph rooted at the node receiving the most NYC-Low cells. NYC-High cells had a higher median within-component pseudotime percentile in eight of nine donors (median donor difference, 0.0791; exact paired signed-rank *P* = 0.0195; BH-adjusted *P* = 0.0547 across the six-configuration comparison; **Figure 6D**). This ordering was used as an exploratory coordinate system for downstream modelling, rather than as evidence of a benign-to-malignant lineage transition.

RegDiffusion followed by SCENIC motif pruning identified 340 regulons in a balanced subset of 5,000 NYC-Low and 5,000 NYC-High malignant cells. Within-specimen NYC-High–NYC-Low differences in regulon activity were adjusted for S-phase and G2M scores and then averaged within donors. Candidate regulators were prioritised by the mean rank of their donor-standardised activity difference and the smallest mean difference observed after leaving out one donor. This analysis linked candidate selection to reproducible activity differences within malignant cells, without relying on a benign-to-malignant comparison (**Figure 6E**).

The five highest-ranked eligible regulators were GTF3C2, RARA, MLX, BRF2 and POLR3G. MLX ranked third and was selected for detailed display based on its activity and perturbation results; it was not the highest-ranked regulator. Complete rankings and all simulated candidates are supplied in Supplementary Data R1.

MLX regulon activity remained higher in NYC-High cells after S-phase and G2M adjustment. Removing YBX1 from the 87-target regulon retained 86 targets and preserved the activity difference in all nine patients (mean adjusted AUC difference 0.0231; BH q = 0.00791). Thus, the observed association was not solely attributable to direct inclusion of an NYC-defining gene in the target set.

CellOracle simulations of the prioritised regulators identified opposite mean reference-flow alignments after MLX knockout and overexpression in NYC-High cells (−0.183 and 0.144; BH q = 0.0134 and 0.0201; **Figures 6F–H**). Correction retained the full combined 72-endpoint family from the candidate and historical-reference simulations. These effects describe network-conditioned predictions, not experimentally established dependencies. In a separate association analysis, MLX regulon activity covaried positively with glycolysis and oxidative phosphorylation after adjustment for NYC, G2M, E2F and depth and removal of overlapping MLX targets from pathway sets (effects 0.195 and 0.418; nine of nine patients positive; three-endpoint BH q = 0.00586 for each; **Figure 6I**). The metabolic models do not establish mediation by MLX.

### 2.10 *In silico* CENPF knockout prioritises cell-cycle and chromosome-segregation network responses

We simulated CENPF loss in a regulatory network reconstructed from NYC-High malignant melanocytic cells using scTenifoldKnk (**Figures 7A–C**). Prioritised model responses were concentrated in cell-cycle regulation, chromosome segregation and mitosis, consistent with the established functions of CENPF. These predictions nominate experimental endpoints for testing CENPF perturbation; they do not demonstrate cellular dependency, lethality or malignant-state reversal.

**Figure 7:**
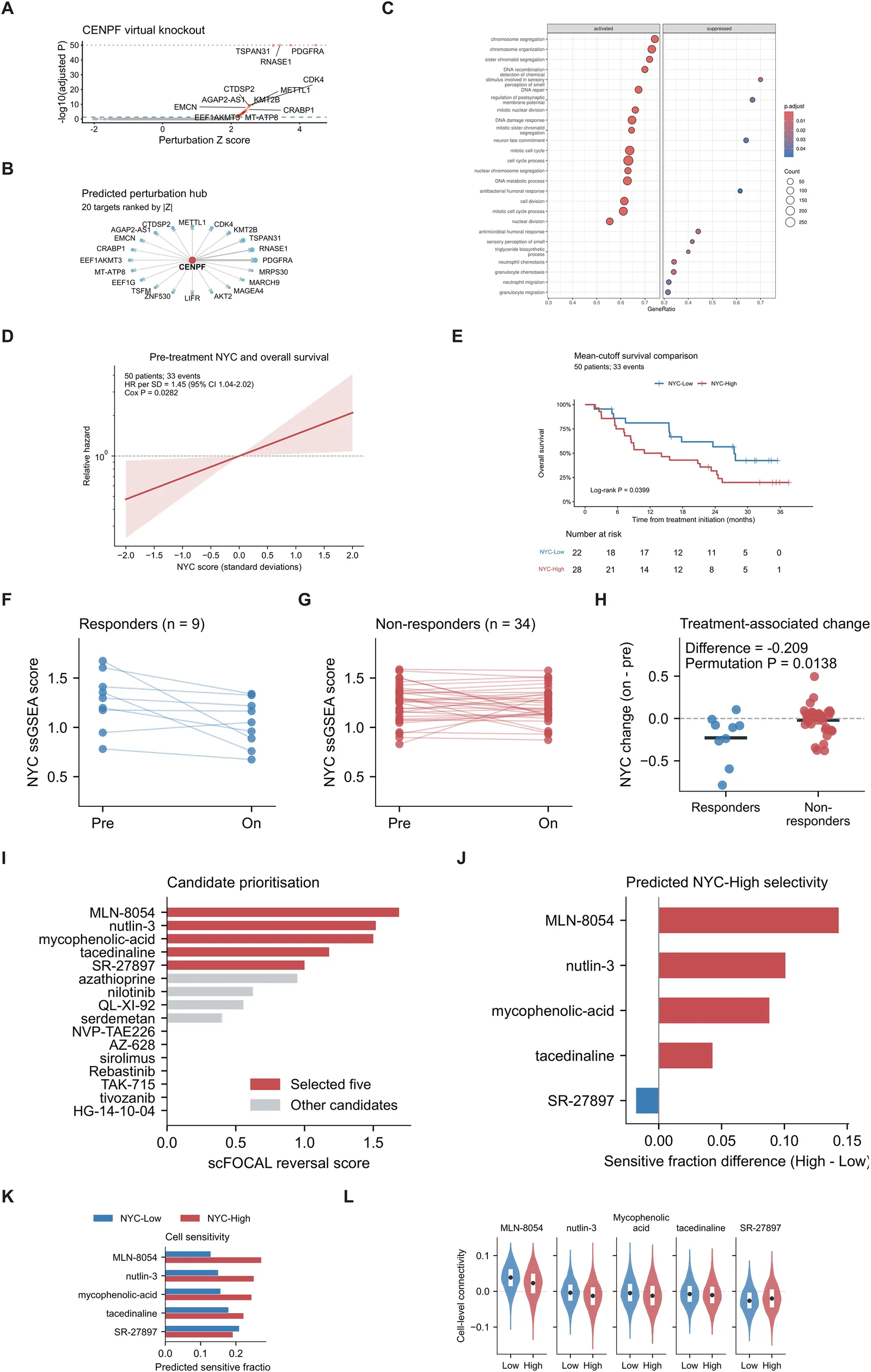
Predicted CENPF network responses, treatment-cohort analyses and computational drug prioritisation. **(A–C)**, scTenifoldKnk-predicted gene responses, response network and Gene Ontology enrichment. **(D)**, Continuous NYC association with overall survival in 50 independent pretreatment GSE91061 patients (33 deaths); line and shading show fitted relative hazard and its 95% interval, referenced to the mean score. **(E)**, Mean-cutoff Kaplan-Meier curves in 50 pretreatment patients (33 deaths) with survival of at least one month; numbers at risk are displayed. **(F, G)**, Paired pre- and on-treatment NYC scores in nine responders and 34 non-responders, respectively; lines join samples from the same patient. **(H)**, Patient-level on-minus-pre NYC changes; horizontal black marks show group means. The displayed P is the two-sided permutation value. **(I)**, scFOCAL prioritisation of DrugReflector candidates; the selected five are highlighted. **(J)**, Difference in predicted sensitive-cell fractions between NYC-High and NYC-Low. **(K)**, Predicted sensitive fractions by state (blue, NYC-Low; red, NYC-High). **(L)**, Cell-level drug-connectivity distributions (blue, NYC-Low; red, NYC-High); white segments span the interquartile range and dark points show medians. Lower connectivity indicates stronger predicted reversal. Clinical filtering and complete sensitivity results are specified in Methods and Supplementary Data R3. Drug panels describe computational predictions, not measured drug efficacy. In B, the same 20 targets ranked by absolute Z score are displayed in a compact radial layout; node size represents absolute Z score and edge width represents the saved regulatory-impact fold change. A retains the original adjusted-P display cap of 50.

### 2.11 Treatment-cohort associations and two-stage compound prioritisation

In GSE91061, the revision-stage pretreatment survival analysis retained 50 independent patients and 33 deaths after excluding one patient with survival below one month. Continuous NYC was associated with overall survival (HR per SD 1.45, 95% CI 1.04–2.02; nominal P = 0.0282; **Figure 7D**); mean-score dichotomisation yielded a log-rank P = 0.0399 (**Figure 7E**). Amongst 43 patients with paired pre- and on-treatment samples, nine responders showed a larger NYC decrease than 34 non-responders (mean changes −0.229 versus −0.020; difference −0.209; bootstrap 95% CI −0.404 to −0.033; permutation P = 0.0138; **Figures 7F–H**). A baseline-adjusted model supported a response-associated change (P = 0.0285), although a Welch comparison gave P = 0.0659. Direct response comparisons and external-cohort results did not establish consistent baseline predictive performance. Treatment-associated dynamics are therefore distinguished from pretreatment response prediction.

We next translated the NYC-High transcriptional state into a therapeutic-prioritisation framework. DrugReflector was first applied to the genome-wide TCGA NYC-High versus NYC-Low signature to identify compounds predicted to reverse the patient-level disease state.

The top-ranked candidates were standardised and mapped to L1000 perturbation profiles, after which scFOCAL-based single-cell connectivity analysis evaluated both transcriptional reversal and state selectivity in NYC-High and NYC-Low malignant cells. Of the mapped candidates, integrated ranking prioritised MLN-8054, mycophenolic acid, nutlin-3, tacedinaline and SR-27897. Compound prioritisation is shown in **Figure 7I**.

MLN-8054, mycophenolic acid and nutlin-3 showed the strongest combined evidence of predicted state reversal and preferential sensitivity within the NYC-High population. These results nominate experimentally testable compounds rather than establishing drug efficacy, as the ranking remains dependent on perturbation-library coverage, cellular context and computational connectivity assumptions. State-selectivity contrasts, sensitive-cell fractions and cell-level connectivity distributions are displayed in **Figures 7J–L**, respectively.

## 3 Discussion

This study connects patient-matched m^5^C-MeRIP and RNA observations with NSUN5 perturbation and malignant-cell expression states. Paired edgeR analysis identified increased relative CENPF IP enrichment at two regions, whilst NSUN5 knockdown reduced CENPF m^5^C enrichment and protein abundance. Patient-aware malignant-cell analyses linked NYC to an immune-resistance transcriptional programme after proliferation adjustment, and nominated MLX for mechanistic follow-up. Treatment-associated NYC changes provided an additional clinical context, without establishing baseline predictive performance. Together, these findings support an m^5^C-associated programme rather than a fully resolved NSUN5–YBX1–CENPF pathway.

An important starting point of this study was to extend *CENPF* dysregulation beyond altered gene expression to the epitranscriptomic level. Through the action of reader proteins, m^5^C can influence mRNA stability, nuclear export and translational efficiency, and *YBX1*-mediated, m^5^C-dependent transcript stabilisation has been mechanistically demonstrated in multiple biological models (14–16). The cooperative activity of *NSUN2* and *YBX1* in breast cancer further illustrates how an m^5^C writer and reader can jointly sustain cancer-associated mRNAs (19). In our data, the positive correlation between *YBX1* and *CENPF*, the concordant expression patterns of the NYC genes in tissues and cellular models, and the parallel reductions in *CENPF* m^5^C enrichment and protein abundance following *NSUN5* perturbation collectively support a coherent writer–candidate reader–effector transcript model in melanoma. The established roles of *NSUN5* in ribosome biology and stress adaptation provide a biological foundation for this model (20, 21). Its reported involvement in tumour immune evasion and in the progression of renal and liver cancers suggests disease-related functions extending beyond constitutive ribosomal RNA modification (22–24). Direct evidence that *NSUN5* can regulate cancer-associated mRNAs through m^5^C modification further supports its plausibility as the candidate writer in this model (25).

CENPF has established roles in late G2, mitosis and chromosome segregation (26–29). Its contribution to NYC makes proliferation an important alternative explanation. Our proliferation-adjusted survival analyses did not establish independent prognostic value. The retained malignant-cell immune-programme association addresses a different question: whether expression states differ beyond measured proliferation within patients. Predicted responses to CENPF perturbation provide hypotheses about associated networks, not experimental evidence of dependence.

Recent melanoma biomarker frameworks distinguish risk stratification, treatment selection and longitudinal monitoring, and emphasise context-specific validation before clinical implementation (37). A prognostic biomarker describes outcome risk, whereas a treatment-predictive biomarker identifies differential benefit from a particular therapy relative to an appropriate comparator; an association in a single treated cohort does not establish this distinction (38). Accordingly, the pretreatment NYC survival association is prognostic within the treated GSE91061 cohort, not evidence of treatment-specific benefit. The paired NYC changes are treatment-associated observations, not an established baseline predictor of ICI response. The small responder subgroup and variation across statistical approaches make these findings exploratory and motivate prospective longitudinal validation with an appropriate treatment comparator.

The clinical relevance of NYC-High also extends to its associated tumour ecosystem. Effective antitumour immunity depends on an intact cancer–immunity cycle and the overall architecture of the tumour immune microenvironment (39, 40). Immune-cell identity, density and spatial distribution, together with tumour-intrinsic inflammatory programmes, collectively shape clinical responses (41, 42). In the present study, NYC-High tumours exhibited broadly lower T-cell-inflamed, CD8^+^ effector, natural killer-cell, dendritic-cell and immunotherapy-associated scores in TCGA-SKCM. This pattern is more consistent with an immune-hyporesponsive state than with the selective expansion of a single immunosuppressive population. TGF-β signalling can promote T-cell exclusion and immune evasion (43, 44), whilst extracellular matrix remodelling can alter immune infiltration and tumour behaviour (45). Hypoxic adaptation can likewise facilitate treatment resistance through both tumour-intrinsic and immune-regulatory mechanisms (46, 47). The altered MIF, LAMININ and CD99 networks inferred by CellChat and CommPath, together with the spatial co-occurrence of NYC activity with TGF-β and hypoxia signatures, provide complementary support for a link between high tumour-cell-intrinsic NYC activity and local immune–stromal remodelling. Previous evidence implicating MIF in melanoma progression and immune regulation further strengthens this interpretation (48).

Recent work on terminally exhausted CD8+ T cells highlights that functional state and spatial niche, rather than cell abundance alone, shape antitumour activity (49). NK-cell plasticity under suppressive microenvironmental signals, including TGF-beta, provides a related context for interpreting reduced NK-associated signatures (50). These studies support considering immune-cell states alongside the NYC-associated tissue environment; our expression-based associations do not directly establish terminal exhaustion, NK-cell reprogramming or their causal regulation by NYC.

Single-cell localisation helped prevent bulk-level associations from being mistaken for evidence of tumour-cell specificity. Previous single-cell atlases of melanoma have revealed the complexity of malignant-cell dedifferentiation, T-cell exclusion and treatment-persistent resistant states (30–32). Recurrent phenotypic-switching programmes further indicate that these states represent connected cellular transitions rather than isolated subpopulations (34). In our analysis, multiple scoring algorithms consistently localised high NYC activity to malignant melanocytic cells whilst malignant-only trajectory modelling provided an exploratory reference for comparisons between NYC-Low and NYC-High cells. Against this background, we extended the NYC axis both upstream and downstream rather than restricting the analysis to three-gene co-expression.

Malignant-only regulatory analyses prioritised motif-supported candidates using cell-cycle-adjusted patient effects and leave-one-patient-out stability. MLX ranked third amongst eligible regulators and showed opposing KO/OE reference-flow predictions. Its activity association persisted after excluding YBX1 from its target set, and its links with metabolic expression programmes remained after adjustment for NYC, proliferation and depth. These convergent computational observations justify prioritising MLX for experimental study. They share expression data and model assumptions and should not be described as independent causal validation. Complete rankings and perturbation endpoints are retained in Supplementary Data R1.

The practical value of this framework is to narrow subsequent experiments. YBX1–CENPF binding and functional reader involvement require direct testing, whilst MLX perturbation could test the predicted regulatory and metabolic relationships. Neither the direction of pseudotime nor its alignment with simulated vectors establishes a developmental transition or a linear regulatory cascade.

Spatial analysis added information from tissue neighbourhoods rather than relying solely on inferred cell proportions. Spatial transcriptomics preserves the correspondence between transcriptional signals and their tissue coordinates, allowing local functional ecosystems within tumours to be identified (51, 52). Across four independently derived melanoma sections, NYC activity correlated positively with TGF-β and hypoxia scores, although the magnitude of these associations varied amongst cases. This variation is biologically compatible with the pronounced spatial heterogeneity of melanoma. The findings support a tendency for regions of high NYC activity to occur within stress-adapted and matrix-remodelling microenvironments and complement the metabolic flux alterations predicted by scFEA. Importantly, the spatial results reflect local functional co-occurrence within tissue neighbourhoods rather than assigning each spatial spot to a single cell type.

For therapeutic prioritisation, we adopted a staged strategy comprising patient-level transcriptional signature reversal, mapping to specific drug entities and single-cell prediction of state-associated connectivity. The Connectivity Map and L1000 established the general framework for identifying compounds that reverse disease-associated transcriptional programmes, and pan-cancer studies have shown that the extent of transcriptional reversal can bear an interpretable relationship to drug activity (53–55). Single-cell drug-repurposing methods further enable predicted sensitivity to be localised to disease-relevant cell populations, whilst scFOCAL provides a specific framework for connecting drug perturbations with sensitive and resistant cell states and for prioritising candidate combinations (56–58). MLN-8054 and nutlin-3 nominate hypotheses involving the Aurora A and MDM2–p53 axes, respectively (59, 60). Mycophenolic acid and tacedinaline, in turn, suggest hypotheses related to purine metabolism and histone deacetylation (61, 62). Although these compounds act through distinct molecular nodes, they shared a predicted capacity to reverse the NYC-High transcriptional state. These rankings guide experimental prioritisation but do not distinguish actual state-selective efficacy from general cytotoxicity.

Several interpretive boundaries remain. The discovery cohort comprised only three patient-matched pairs, limiting precision and assessment of heterogeneity. Regional IP/Input enrichment is a relative antibody-enrichment measurement, not a single-base methylation fraction. Endogenous nonpeak normalisation assumes background stability and does not substitute for external spike-in calibration. NSUN5 perturbation supports a functional relationship with CENPF enrichment and protein abundance but does not prove direct catalytic targeting. Published YBX1 reader mechanisms (14–16) motivate its candidacy; we have not demonstrated its direct binding to CENPF or a decrease in CENPF following YBX1 depletion. The proposed reader-mediated relationship is therefore explicitly unconfirmed.

Second, the multiscale bioinformatic analyses primarily establish associations and priorities and do not substitute for causal experiments. The TCGA survival and immune analyses may be influenced by clinical confounders, including disease stage, treatment exposure, tumour purity and sampling site. The three-gene NYC score partially overlaps with cell-cycle activity, and thresholds defining NYC-High and NYC-Low states may be cohort dependent. Cross-study single-cell integration is affected by sample composition, batch-correction procedures and strategies used to identify malignant cells. CellChat, CommPath, scFEA, SCENIC, CellOracle and scTenifoldKnk all depend on reference networks, model assumptions or both. Although spot-level spatial co-occurrence preserves information on tissue neighbourhoods, it cannot resolve causal direction at single-cell resolution. DrugReflector and scFOCAL are additionally constrained by the coverage of their perturbation libraries, drug-name mapping and the cellular backgrounds in which perturbations were profiled. The prioritised compounds should therefore be interpreted as computational candidates for experimental testing rather than as evidence of clinical efficacy. The M05 trajectory was chosen after exploratory parameter comparisons; nominal patient-level significance is not independent confirmation of an optimised developmental direction. The GSE91061 one-month survival restriction was also introduced during revision and is not a prospectively specified eligibility criterion. Treatment-response conclusions depend on timing, endpoint definition and statistical assumptions; complete comparisons are supplied rather than treating selected results as independent replication.

The study supports the NYC programme as an m^5^C-associated framework for describing melanoma cell states and generating mechanistic and therapeutic hypotheses. Its value lies in linking patient-derived molecular observations and *NSUN5* perturbation findings to cellular and tissue context, rather than establishing a fully resolved linear pathway or a clinically validated treatment-selection marker.

## 4 Materials and methods

### 4.1 Study design

The study comprised four stages: discovery, validation, mechanistic extension and therapeutic prioritisation. The discovery stage used joint m^5^C-MeRIP-seq and RNA-seq profiling of three patient-matched pairs of melanoma and normal tissues. Clinical validation integrated TCGA-SKCM, public normal-skin and melanoma expression resources, multiple survival cohorts and GSE91061. Experimental validation comprised in-house immunohistochemistry, baseline western blotting in cellular models, siRNA perturbation, *CENPF*-targeted MeRIP–qPCR and post-perturbation western blotting. Mechanistic extension incorporated single-cell RNA sequencing, CellChat, CommPath, scFEA, spatial transcriptomics, Monocle3, SCENIC/RegDiffusion, CellOracle and scTenifoldKnk. Therapeutic screening was performed sequentially using DrugReflector and scFOCAL/L1000-based single-cell connectivity analysis.

### 4.2 m^5^C-MeRIP-seq and matched RNA-seq

Three melanoma tissues (A1–A3) and their patient-matched normal tissues (B1–B3) were included. After quality assessment, total RNA was fragmented to approximately 200 nucleotides. m^5^C-modified RNA was immunoprecipitated using the GenSeq m^5^C MeRIP Kit, with corresponding input fractions retained. Libraries were subjected to paired-end sequencing on the BGI T7 platform. Adapters and low-quality reads were removed using Cutadapt v1.9.3, after which reads were aligned to the human hg38 reference genome using HISAT2 v2.0.4. m^5^C peaks were called using MACS v1.4.2, and the initial vendor differential-peak screen used diffReps v1.55.6 with an FDR threshold of 0.05. The paired regional reanalysis described below supersedes those screening statistics for inference at P1–P3. The six Input libraries also provided gene-level RNA expression measurements and were not counted as an additional independent sequencing cohort. MeRIP-seq and MeRIP-qPCR were performed by Cloudseq Biotech Inc. (Shanghai, China). The company also performed primary sequencing-data processing.

Paired regional reanalysis was performed in R 4.3.2. Twelve libraries represented six tissue specimens from three patients, with A1–B1, A2–B2 and A3–B3 matched within patient. Paired fragments were counted with featureCounts 2.1.1 using reverse-strand specificity, primary alignments, MAPQ ≥10 and exclusion of multimapping fragments. Fragments overlapping multiple features contributed integer counts to each eligible feature; no additional PCR-duplicate removal was performed. Candidate regions were the strand-specific union of the six single-sample peak sets (11,747 regions). Regions with pooled Input counts >30 and pooled IP counts >30 were retained, yielding 7,937 candidates. P1–P3 matched three union regions exactly and were not duplicated in the testing family.

Normalisation used nonpeak exonic intervals after removing candidate and original target intervals. The background contained 299,002 bins of 100–1,000 nt; DESeq2 median-ratio factors were estimated from the 104,980 bins with positive counts in every library and centred to geometric mean one. These background factors were supplied as library offsets rather than calculated from CENPF alone. edgeR 4.0.16 used robust dispersion estimation and quasi-likelihood F testing (63). The design contained six tissue baselines, an IP effect, patient-specific IP effects and the condition-by-IP interaction (rank 10 across 12 libraries; nominal residual degrees of freedom 2). The interaction estimates relative IP/Input change, not RNA expression fold change or absolute methylation fraction. BH adjustment covered all 7,937 retained regions. No sample or candidate was removed on the basis of its effect or P value.

Paired Input RNA expression was analysed separately using DESeq2 1.42.1 with patient and tissue-condition covariates. Genes with at least ten total counts were retained (28,208 genes); independent filtering was disabled for BH adjustment. Regional IP enrichment and gene-level expression are distinct endpoints. The edgeR analysis was originally specified as a model sensitivity analysis and was selected for presentation during revision; it is not described as prospectively registered. Supplementary Data R2 provides count, design and complete edgeR output files.

### 4.3 *CENPF* m^5^C peak, metagene and motif analyses

Peaks annotated to the *CENPF* transcript ENST00000366955 were extracted from the MACS output. The peak-call burden was calculated for each sample, and sample-resolved tracks were plotted using a common set of genomic coordinates. The vendor differential table was used for initial *CENPF* target nomination. Formal regional effect estimates and adjusted *P* values in the revision were taken from the paired Input-adjusted analyses above, using the full candidate family rather than a *CENPF*-only testing family. Transcriptome-wide m^5^C peaks were mapped to relative positions within the 5′ untranslated region (5′ UTR), coding sequence (CDS) and 3′ untranslated region (3′ UTR) to calculate normalised metagene density. Peak sequences from the two tissue groups were separately analysed using DREME to identify enriched short-sequence motifs. The recurrence of P1–P3 was summarised from the enrichment intensity and detection status of the corresponding regions in each sample.

### 4.4 RNA expression and *YBX1*–*CENPF* correlation

The fold change, nominal *P* value and FDR for *CENPF* were obtained from the gene-level differential-expression analysis of the matched RNA-seq data. The association between *YBX1* and *CENPF* expression across the six sequenced specimens was assessed using a two-sided Spearman rank-correlation test, with Spearman’s *ρ* and the corresponding *P* value reported. Patient matching was used only for within-cohort comparisons in the discovery dataset; these samples were not pooled with public cohorts for correlation analysis.

### 4.5 Public expression, survival and cross-cohort Cox analyses

Differences in *NSUN5*, *YBX1* and *CENPF* expression between normal skin and SKCM were assessed using uniformly normalised public expression matrices and two-sided Wilcoxon rank-sum tests. For single-gene overall-survival analyses, patients were stratified into high- and low-expression groups using gene-specific optimised cut-offs. Survival curves were estimated using the Kaplan–Meier method and compared using two-sided log-rank tests. Univariable Cox proportional-hazards models were fitted separately in multiple public melanoma cohorts, and hazard ratios (HRs) with 95% confidence intervals (CIs) were summarised in forest plots.

### 4.6 TCGA-SKCM NYC score and survival analysis

A preprocessed TCGA-SKCM log_2_(TPM + 1) matrix comprising 19,344 genes and 473 samples was used. NSUN5, YBX1 and CENPF were treated as a fixed gene set, and a weighted single-sample gene-set enrichment analysis (ssGSEA; α = 0.25) was used to calculate the NYC score (64, 65). Samples were divided into NYC-High and NYC-Low groups at the cohort-wide median. Genome-wide differential effects were calculated from the difference in group means, Welch’s t-test and Benjamini–Hochberg FDR. The standardised effect used for drug screening was defined as (meanHigh − meanLow)/√(varHigh + varLow).

Survival analysis included 456 patients with complete overall-survival information. NYC groups were evaluated using Kaplan–Meier curves, two-sided log-rank tests and Cox proportional-hazards models. The proportional-hazards assumption was assessed using Schoenfeld residuals. Analyses were conducted using survival v3.5–7 and survminer v0.5.1.

Proliferation-adjusted Cox models used a common complete-case set of 378 patients (178 deaths; 93 primary and 285 metastatic specimens). Covariates were standardised age, sex, stage (0/I/II versus III/IV), and primary versus metastatic sampling type; G2M and E2F were added separately and together. An additional model stratified the baseline hazard by sampling type. TCGA participant IDs were defined by the first 12 barcode characters; where multiple specimens occurred, primary (01), metastatic (06), then additional metastatic (07) were prioritised, with lexical sample order breaking ties. This rule was not outcome selected. Clinical sampling type is not equivalent to stage or time from diagnosis. All model coefficients, proportional-hazards diagnostics and score correlations are supplied in Supplementary Data R1.

### 4.7 Tumour-microenvironment and immunotherapy-related signatures

Within the same TCGA NYC stratification, IOBR v0.99.8 was used to calculate tumour-microenvironment cell-type features, immune-exclusion and immunosuppression signatures, and immunotherapy-related biomarker scores (66). Between-group comparisons used two-sided Wilcoxon rank-sum tests, with Benjamini–Hochberg correction applied within each functional module. Results were interpreted jointly on the basis of effect direction, score distribution and adjusted significance.

### 4.8 Immunohistochemistry and whole-slide imaging

Paraffin-embedded tissue sections were deparaffinised, rehydrated, subjected to antigen retrieval and treated to block endogenous peroxidase activity. Sections were then incubated separately with primary antibodies against *NSUN5*, *YBX1* and *CENPF*, followed by horseradish peroxidase-conjugated secondary antibodies, 3,3′-diaminobenzidine development and haematoxylin counterstaining. Slides were scanned at ×20 using an LG-S80 digital pathology scanner at a spatial resolution of 0.273810 μm per pixel, using the immunohistochemistry calibration mode for whole-slide digitisation. **Figure 3A** presents representative high-magnification fields traceable to the original whole-slide images and covering normal skin, benign naevi and melanoma. Complete whole-slide overviews are provided in **Supplementary Figure S2**.

### 4.9 Cell culture and siRNA perturbation

The 451Lu melanoma cell line was kindly provided by Professor Chunying Li, Xijing Hospital, China. HEMa, A375 and A2058 cells were obtained from iCell Bioscience Inc., Shanghai, China. Normal human epidermal melanocytes (HEMa) and the human melanoma cell lines A375, A2058 and 451Lu were maintained at 37 °C in 5% CO_2_ according to supplier recommendations and standard laboratory procedures. A375 and A2058 cells were transfected with three independent siRNAs targeting NSUN5 and the corresponding negative controls. Cells were collected at prespecified time points for western blotting and CENPF-targeted MeRIP–qPCR. The three siRNAs were initially screened according to the reduction in NSUN5 protein abundance, and a working perturbation condition showing a consistent directional effect was used for subsequent experiments.

### 4.10 Western blotting and densitometric quantification

Cells were lysed in RIPA buffer on ice for 30 min. Lysates were centrifuged at 12,000 rpm for 10 min at 4 °C, and the supernatants were collected. Total protein concentration was measured using the bicinchoninic acid assay. Equal amounts of protein were separated by SDS–PAGE and transferred to 0.45-μm PVDF membranes at 350 mA for 30 min. Membranes were blocked with 5% non-fat milk for 30 min and incubated with primary antibodies overnight at 4 °C. After washing with TBST, membranes were incubated with horseradish peroxidase-conjugated secondary antibodies for 30 min. Signals were developed with enhanced chemiluminescence for 1 min and acquired using an SCG-W3000 imaging system.

Primary antibodies against NSUN5, YBX1, CENPF, GAPDH and HSP90 were used. The rabbit anti-CENPF antibody was HuaAn HA721508 (1:1,000), paired with HRP-conjugated goat anti-rabbit IgG (Servicebio GB23303, 1:3,000). The mouse anti-HSP90 antibody was Servicebio GB15284 (1:700), paired with HRP-conjugated goat anti-mouse IgG (Servicebio GB23301, 1:3,000). Densitometry was performed using AIWBwell, and target-protein abundance was normalised to the corresponding loading control. Baseline-expression experiments comprised three independent biological replicates and were analysed by one-way analysis of variance followed by Dunnett’s test using HEMa as the common control. Changes in CENPF protein abundance after siNSUN5 treatment were analysed using three paired biological replicates and two-sided paired t-tests. Bar plots show the mean ± s.d. together with individual replicate values.

### 4.11 *CENPF*-targeted MeRIP–qPCR

Total RNA was prepared at a concentration of approximately 1 μg μl^−1^, and 18 μg RNA was used per reaction. RNA was heated at 70 °C for 3 min to generate fragments of approximately 200 nucleotides. A 3-μg aliquot was retained as input, and the remaining RNA was immunoprecipitated using the GenSeq m^5^C MeRIP Kit. Each reaction contained 25 μl PGM beads and 2 μl m^5^C antibody and was incubated for 1 h at 4 °C. RNA was then eluted and digested for 45 min at 55 °C before purification. Reverse transcription was performed using SuperScript III and random N6 primers at 50 °C for 60 min followed by 70 °C for 15 min. Quantitative PCR was carried out using GenSeq SYBR on a QuantStudio 5 instrument in a 10-μl reaction volume. Cycling conditions were 95 °C for 10 min, followed by 40 cycles of 95 °C for 10 s and 60 °C for 60 s.

Primers targeting the *CENPF* P1 region were forward 5′-GCTGCGGGCAGTTTGAATTAG-3′ and reverse 5′-GACGGGAAAACGACCTCTCG-3′, generating an 86-bp amplicon. Three technical replicates were included for each sample. Enrichment was calculated as %IP/Input = 2^(*CtInput* − *CtIP*) × dilution factor × 100, with an input dilution factor of 1/10. Statistical analysis was based on log_2_(% input) values from three independent biological replicates, using a two-sided Welch’s *t*-test.

### 4.12 Single-cell data processing and integration

The single-cell datasets comprised three benign-naevus samples from GSE277165 and ten melanoma samples from GSE215120; the original studies were reported by Stubenvoll et al. and Zhang et al., respectively (67, 68). Ambient RNA contamination was estimated separately for each sample using DecontX (69). Cells were retained if they had >200 and <6,000 detected features, a mitochondrial-transcript proportion <15% and an estimated contamination score <0.25. Doublets were identified and removed on a per-sample basis using scDblFinder (70). To reduce potential bridge cells with concurrent high melanoma-marker and immune-marker expression, an additional filtering step was applied using predefined module scores.

Retained cells were normalised using Seurat LogNormalize (71, 72), and 2,000 highly variable genes were selected. Mitochondrial, ribosomal, haemoglobin and immediate-early response genes were excluded from the variable features. ScaleData was used to regress percent.mt, contamination, S.Score and G2M.Score. Principal-component analysis was performed using 50 components. Harmony was applied with sample as the batch variable (73) (theta = 1; max.iter.harmony = 20). A nearest-neighbour graph was constructed using the first 50 Harmony dimensions, followed by clustering at a resolution of 1. UMAP parameters were n.neighbors = 30, min.dist = 0.2, spread = 2 and metric = cosine. The final integrated object comprised 79,133 cells and 38,224 genes.

Integration sensitivity analyses retained all 79,133 cells, original annotations and 1,986 original PCA features. batchelor 1.18.1 fastMNN (74) used stored log-normalised RNA, sample batches, k = 20, d = 50, cosine normalisation, ndist = 3, fixed merge order and seed 42. A separate reduced MNN run used the historical regressed PCA coordinates to match preprocessing. Standard fastMNN and historical Harmony therefore did not have identical preprocessing. Neighbour diagnostics used 30 neighbours in 50 dimensions, not UMAP distances. Cross-study mixing summaries excluded the confounded melanocytic populations. These analyses assess integration sensitivity, not the identifiability of benign–malignant effects (**Supplementary Figure R1**; **Supplementary Data R1**). For **Supplementary Figure R1**, all four embeddings were newly projected with a common UMAP setting (50 dimensions, 30 neighbours, min.dist = 0.2, spread = 1, cosine metric, seed 42 and 500 epochs). These comparison projections are not the historical UMAP displayed in **Figure 4E**, whose saved RunUMAP command used spread = 2. The historical Harmony label in the comparison identifies the input embedding, not reuse of the original two-dimensional coordinates. Neighbour diagnostics were calculated in 50 dimensions and do not depend on the UMAP spread parameter.

### 4.13 Cell annotation and identification of malignant melanocytic cells

Cell types were annotated using canonical markers, cluster-specific marker genes and sample origin. inferCNV was subsequently applied to melanocytic cells to assist in identifying malignant cells from large-scale chromosomal expression shifts (75). B cells, NK cells, CD4^+^ and CD8^+^ T cells, and regulatory T cells were used as reference populations. Parameters were cutoff = 0.1, cluster_by_groups = TRUE, k_obs_groups = 3, HMM = FALSE, denoise = TRUE and num_threads = 8. Malignant melanocytic cells were identified by integrating melanocytic markers, sample origin, large-scale chromosomal shifts and the derived CNV score.

For **Figures 4G–I**, the existing melanocytic cell selection and annotations were retained, and raw UMI counts were retrieved from the original 10x matrices and summed over cells within each specimen. Both malignant NYC states were pooled. The comparison used all three benign-naevus specimens from GSE277165 and one specimen from each of nine melanoma patients from GSE215120; AM3_pre was retained and the repeated AM3_post specimen was not counted as an additional independent replicate. No low-yield specimen was removed on the basis of expression results; one benign specimen contained 36 selected cells. edgeR v4.0.16 used filterByExpr, TMM normalisation, robust dispersion estimation and a quasi-likelihood model with group as the predictor. Displayed log2 CPM values used a prior count of 1. Two-sided tests compared malignant with benign specimens; BH correction in G-I covers the three prespecified programme genes, not all tested genes. Genome-wide FDR, sample-level counts, within-GSE277165 analyses using the saved permissive and CNV-strict selections, and reproduction code are supplied in **Supplementary Data R4**. Dataset and group cannot be jointly identified in the original comparison, so patient-level aggregation does not remove study-origin confounding.

### 4.14 NYC programme scoring and single-cell state definition

The NSUN5/YBX1/CENPF programme was evaluated using AUCell v1.24.0 (76), UCell v2.9.0 (77), singscore v1.22.0 (78), ssGSEA implemented in GSVA v1.50.5 (64), z-score-based scoring and Seurat AddModuleScore. Scores from each method were standardised to a common direction at the cell-type level, and concordance across algorithms was evaluated. The integrated NYC score was defined as the mean of the six method-specific scores. Amongst 46,194 malignant melanocytic cells, the global mean of the integrated NYC score was used to define NYC-High and NYC-Low states for subsequent state-level analyses.

Additional patient-aware state analyses used specimen-level models adjusted for Hallmark G2M, E2F and transcript depth, then averaged specimen effects within patients. Gene-set membership, scaling, all tested endpoints and proliferation-gene-exclusion analyses are supplied in **Supplementary Data R1**. Exact sign-flip tests enumerated the 512 sign patterns of nine patient effects; correction families are specified per table. MLX metabolic models additionally adjusted for NYC and removed overlapping MLX targets from pathway sets; three metabolic endpoints were retained. These are exploratory analyses of the same malignant cohort, not independent validation cohorts.

### 4.15 CellChat analysis

Only cells from melanoma samples were retained, and a CellChat v1.6.1 object was created using cell type as the grouping variable (79). The complete CellChatDB.human database was used, including Secreted Signaling, ECM–Receptor and Cell–Cell Contact categories. The analysis sequentially applied subsetData, identifyOverExpressedGenes, identifyOverExpressedInteractions, projectData(PPI.human), computeCommunProb(raw.use = TRUE), filterCommunication(min.cells = 10), computeCommunProbPathway, aggregateNet and network-centrality analysis. MIF, LAMININ and CD99 were prioritised from the full pathway set on the basis of NYC-High/NYC-Low differences and biological relevance to the immune–stromal ecosystem. Circular communication plots, signalling-role heatmaps and ligand–receptor chord diagrams were generated for each pathway.

### 4.16 CommPath analysis

CommPath v1.1.0 was applied to the Seurat RNA data layer from melanoma samples, using cell type as the cell label (80). The downsampling limit was set to 50,000 cells. Ligand–receptor markers were identified using the Wilcoxon test, with findLRpairs thresholds of logFC = 0 and P = 0.01. KEGG was used for intracellular-pathway mapping, UCell for pathway scoring, and the minimum gene-set size was 10. After diffAllPath(only.posi = TRUE, only.sig = TRUE), significant ligand–receptor/pathway relationships were filtered to generate a global network, an NYC-High-centred chained network and a cell-type pathway-activity heatmap.

### 4.17 Spatial transcriptomic signature scoring and local correlation analysis

Spatial analysis included four melanoma Visium sections for which tissue images and spot coordinates had been verified. Sections 1 and 2 were GSM9459774 (mel2) and GSM9459775 (mel3) from GSE316760. Section 3 was the 10x Genomics Human Melanoma, IF Stained (FFPE) demonstration dataset generated using Visium CytAssist (slide V42A20-355, area A1). Section 4 was the melanoma brain-metastasis sample GSM5420750 (patient 16) from GSE179572 (81, 82). Tissue-coordinate interpretation followed established spatial-transcriptomic principles (51, 52). TGF-β and hypoxia gene sets were HALLMARK_TGF_BETA_SIGNALING and HALLMARK_HYPOXIA from MSigDB Hallmark (83). The NYC score was calculated by aggregating the standardised expression of detectable NSUN5, YBX1 and CENPF transcripts within each section. All scores were standardised within sections before mapping to the original spatial coordinates.

Two-sided Spearman correlations between NYC and TGF-β or hypoxia scores were calculated separately within each section. For local-neighbourhood analysis, a seven-nearest-neighbour graph was constructed from high-resolution spatial coordinates. After excluding the index spot, the mean functional score of its six neighbours was calculated. NYC-high and NYC-low spots were defined by the upper and lower quartiles of the NYC score, respectively, and their surrounding local functional environments were compared. R analyses used msigdbr v25.1.1 and GSVA v1.50.5. Spatial-object processing and visualisation used Python v3.10.20, Scanpy v1.11.5, AnnData v0.11.4, Squidpy v1.6.5, SpatialData v0.5.0, SciPy v1.15.3 and scikit-learn v1.7.2.

### 4.18 scFEA metabolic-flux prediction

A fixed random seed was used to sample 3,000 malignant melanocytic cells, and their raw expression matrix was exported. scFEA was run according to the published framework (84), using source-code Git commit 4c1fb76d52f07bafad84ce7686ad7c3acfcf0126 and the human 168-module model files module_gene_m168.csv, cmMat_c70_m168.csv and cName_c70_m168.csv. sc_imputation was set to FALSE, and cell-level flux and balance matrices were generated. The internal model seed was 16. Differences in module flux between NYC-High and NYC-Low cells were used for ranking and visualisation.

### 4.19 Monocle3 trajectory analysis

Trajectory inference used all 46,194 malignant melanocytic cells from GSE215120, with benign melanocytes excluded. Raw counts were used to construct a Monocle3 v1.4.26 cell data set (85, 86). The frozen M05 configuration used 30 existing Harmony dimensions and Seurat UMAP with 50 neighbours, a minimum distance of 5, a spread of 5, cosine distance and seed 42. The corresponding PCA and UMAP coordinates were imported into Monocle3. Cells were clustered with k = 20 and seed 42, and a principal graph was learned with partitioning and loop closure enabled. Within each graph component, the node receiving the largest number of projected NYC-Low cells was selected as the root. Pseudotime was converted to a within-component percentile. Sample–component comparisons required at least 30 cells with finite pseudotime in each NYC state. Median NYC-High–NYC-Low differences were summarised across eligible components within specimens and then across specimens within donors. The two AM3 specimens were treated as repeated observations from one donor, giving nine donor-level effects. Two-sided exact signed-rank tests and BH correction across the six-configuration comparison were reported. Fixed-graph leave-one-donor-out and AM3-post exclusion checks were retained. M05 was selected for descriptive downstream modelling after exploratory parameter comparisons; these analyses do not constitute independent validation of a developmental direction.

### 4.20 RegDiffusion/SCENIC regulatory-network inference

Regulatory-network inference used 10,000 malignant cells, comprising 5,000 NYC-Low and 5,000 NYC-High cells sampled across the 10 specimens with seed 20260907. No benign melanocytes were included. Three thousand highly variable genes were combined with detectable transcription factors, retaining 4,523 genes after variance filtering, including 1,759 transcription factors. RegDiffusion v0.2.2 in OmicVerse v2.2.3 under Python v3.10 was used for network inference, followed by SCENIC co-expression module construction, motif pruning and AUCell activity estimation (76, 87). RegDiffusion used 300 optimisation steps, a batch size of 64, a learning rate of 0.001, a sparsity-loss coefficient of 0.25 and seed 42. Motif pruning used the hg38 RefSeq-r80 cisTarget gene–motif ranking database spanning 10 kb upstream and downstream of transcription start sites (mc9nr) and the corresponding v9 HGNC motif annotations. Eight workers were used, with dropout masking enabled, module thresholds of 0.75 and 0.9, the top 50 targets, and the top 5, 10 and 50 regulators. The resulting 340 regulons were scored in all 10,000 cells. Dependencies included Scanpy v1.11.5, AnnData v0.11.4, PyTorch v2.12.1, NumPy v2.2.6 and pandas v2.3.3.

### 4.21 Donor-aware prioritisation of candidate transcription factors

For each specimen and regulon, AUCell activity was modelled as a function of NYC state and standardised S-phase and G2M scores. The NYC-High coefficient was extracted and averaged across specimens from the same donor. Two-sided exact sign-flip tests were performed on the nine donor effects, with BH correction across 340 regulons; unadjusted analyses and a combined 680-test correction were retained as sensitivity analyses. Eligible regulators had at least 20 motif-supported targets and expression detected in at least 1% of cells; the three NYC-defining genes were excluded as regulator candidates. Priority was the equal-weight mean percentile rank of the donor-standardised adjusted effect and the minimum mean effect after leaving out one donor. The five highest-ranked unique eligible regulators were selected before perturbation results were examined. A historical reference candidate was also analysed and retained in the complete results, but MLX is the focus of the revised figure. To assess dependence on score-defining genes, NSUN5, YBX1 and CENPF were removed from regulon target sets where present, followed by repeat AUCell scoring, donor-level adjustment and BH correction.

### 4.22 CellOracle *in silico* perturbation

CellOracle v0.18.0 was applied to the same 10,000 malignant cells, with the M05 embedding and pseudotime retained (88). The current motif-pruned regulon edges were used to construct a new base regulatory network. Library sizes were calculated from all measured genes; expression was scaled to 10,000 counts per cell and log-transformed once by the CellOracle importer. Genes detected in fewer than three cells were removed, leaving 4,217 genes. K-nearest-neighbour imputation used 30 principal components and k = 250. Separate NYC-Low and NYC-High regulatory links were inferred with ridge α = 10 and 20 bagging iterations, filtered at P < 0.001, and restricted to the top 10,000 links by absolute coefficient per state. Cluster-specific regulatory models were fitted with α = 10. The five prioritised regulators and a historical reference candidate were each subjected to KO and OE simulation, yielding 12 runs.

KO set target-gene expression to zero, whereas OE used the 95th percentile of the gene-specific imputed expression distribution. Simulated changes were propagated for three steps with clipping enabled. Transition estimation used 200 neighbours, a sampled fraction of 1 and seed 42; embedding shifts used a correlation bandwidth of 0.05. Reference and simulated vector fields were evaluated on a 40 × 40 grid with smoothing of 0.8, 200 neighbours and a minimum mass of 0.01. Flow inner products were assigned to cells from the nearest retained grid point. Cell-level metrics were averaged within specimen and NYC state, then within donor. Two-sided exact sign-flip tests used all 512 sign patterns for the nine donor effects. BH correction covered the 60 signed endpoints from the five primary candidates, with 12 historical-reference endpoints reported separately and a combined 72-endpoint sensitivity correction. Non-negative shift magnitudes were described without a zero-null significance test. The filtered network did not model NSUN5, so no incomplete three-gene NYC perturbation score was constructed. These statistics describe donor-aggregated responses conditional on a shared fitted network and exploratory reference ordering. Additional dependencies included Scanpy v1.10.3, AnnData v0.10.8 and NumPy v1.26.4.

### 4.23 *CENPF in silico* knockout using scTenifoldKnk

Using seed = 123, 3,000 NYC-High cells were sampled. Five thousand highly variable genes were selected, with CENPF forcibly retained, and a single-cell gene-regulatory network was constructed from raw counts. CENPF knockout was simulated using scTenifoldKnk v1.0.3 (89). Significant responses were defined by Benjamini–Hochberg FDR < 0.05 and |Z| > 1.5. Network objects were processed using scTenifoldNet v1.3, and Gene Ontology enrichment was performed using clusterProfiler v4.10.1 (90) and org.Hs.eg.db v3.18.0.

### 4.24 GSE91061 treatment-cohort survival analysis

GSE91061 pretreatment survival analyses included 50 independent patients with 33 deaths, retaining one pretreatment expression profile per patient. During revision, one patient recorded as dying 10 days after immunotherapy initiation was excluded using a survival threshold of 30.4375 days; this exclusion was not prespecified. The exploratory rationale was to examine the association beyond very early mortality, because 10 days precedes the usual response-assessment interval: immune-related response criteria recognise delayed responses, and iRECIST generally recommends reassessment every 6–12 weeks (91, 92). These response-assessment recommendations do not prescribe a one-month exclusion from overall-survival analyses, and an early death remains a valid survival event. The restriction therefore conditions on observed survival and may introduce selection bias. NYC ssGSEA used the fixed three-gene set and was standardised for continuous Cox analysis. Proportional hazards were assessed with Schoenfeld residuals. Kaplan-Meier curves used the mean NYC score calculated within the retained analysis set. Survival time remained measured from treatment initiation, so this is not a formal landmark analysis. Paired treatment dynamics used all 43 eligible pre/on-treatment patients without applying this survival filter. Objective responders were CR/PR and non-responders SD/PD. Differences in on-minus-pre NYC change were assessed by two-sided label permutation and group-stratified bootstrap intervals, with Welch and baseline-adjusted ANCOVA checks. Two-score BH and the wider historical testing-family correction are retained in **Supplementary Data R3**. Baseline, on-treatment and external response analyses are presented separately and overlapping resources are not counted as independent replications.

### 4.25 DrugReflector bulk signature-reversal analysis

A disease signature was constructed from the genome-wide standardised differential effects of NYC-High versus NYC-Low tumours in TCGA. This approach was based on the Connectivity Map/L1000 concept of reversing disease-associated transcriptional states (53–55). The signature was entered into DrugReflector v1.0.0 in the reverse direction, such that higher output scores indicated greater predicted potential to reverse the NYC-High state. Officially released model weights and compound resources were used (Zenodo DOI: 10.5281/zenodo.16912444) (58). The top 500 candidates ranked by DrugReflector score were extracted. Drug names were standardised using perturbagen identifiers, canonical names, case-insensitive matching, hyphenation, salt forms and common synonyms. The top 20 named candidates were retained for database mapping.

This module used Python v3.14.0, PyTorch v2.12.0+cpu, NumPy v2.4.2, pandas v2.3.3, SciPy v1.16.3, scikit-learn v1.8.0, matplotlib v3.10.8 and seaborn v0.13.2.

### 4.26 scFOCAL/L1000-based single-cell drug-connectivity validation

The second-stage screen used R v4.3.2, Seurat v5.5.0, scFOCAL v0.1.0 and MAST v1.28.0 (56, 57, 93). With random seed 20260610, up to 5,000 malignant cells were sampled from each of the NYC-High and NYC-Low states. MAST was used to construct a High-versus-Low differential-expression signature. Intersecting this signature with the L1000 drug-perturbation matrix yielded 865 shared genes. For 16 DrugReflector candidates that were successfully mapped, the numbers of oppositely and concordantly regulated genes and a reversal score were calculated. Cell-level connectivity was defined by the Spearman correlation between each cell’s expression profile and the drug-perturbation signature.

For each drug, cells in the lowest 20% of the overall connectivity distribution were classified as predicted sensitive, those in the highest 20% as predicted resistant, and the remainder as intermediate. The same drug-specific thresholds were applied to NYC-High and NYC-Low cells. Connectivity distributions were compared using two-sided Wilcoxon rank-sum tests. Candidate compounds were jointly ranked by reversal score, the predicted sensitive fraction in NYC-High cells and the difference in sensitive fractions between NYC-High and NYC-Low cells. The five highest-ranked compounds were examined in greater detail at the cell-state level.

### 4.27 Statistical analysis and visualisation

Continuous variables were analysed using Welch’s t-test, paired t-test, Wilcoxon rank-sum test, Kruskal–Wallis test or Spearman rank correlation, as appropriate to the experimental design. Comparisons of multiple treatment groups against a common control used Dunnett’s procedure. Pseudobulk gene contrasts used edgeR quasi-likelihood tests with the three-gene and genome-wide BH families reported separately. High-dimensional gene, regulon, network and enrichment analyses used Benjamini–Hochberg FDR correction. Survival analyses report log-rank P values and, where applicable, Cox HRs with 95% CIs. Western blot quantification is shown as the mean ± s.d. together with independent biological replicates. Fixed random seeds were used for all random sampling and computational perturbations. Regional IP/Input enrichment and Input RNA expression were analysed as distinct endpoints. Regional BH adjustment retained the full set of 7,937 eligible candidates, without restricting correction to CENPF. Patient-aware analyses explicitly distinguish patients from specimens and cells; exploratory analyses are not presented as prospectively validated.

The principal R software packages were R v4.3.2, Seurat v5.5.0, SeuratObject v5.0.2, SingleCellExperiment v1.24.0, celda v1.18.2, scDblFinder v1.14.0, harmony v1.2.3, SCP v0.5.6, infercnv v1.18.1, Matrix v1.6-5, BiocParallel v1.36.0, irGSEA v3.2.5, limma v3.58.1, edgeR v4.0.16, MAST v1.28.0, CellChat v1.6.1, CommPath v1.1.0, Monocle3 v1.4.26, scTenifoldKnk v1.0.3, clusterProfiler v4.10.1, GSVA v1.50.5, IOBR v0.99.8, ComplexHeatmap v2.18.0, pheatmap v1.0.13, ggplot2 v4.0.0, patchwork v1.3.2, cowplot v1.2.0, ggpubr v0.6.1 and ggrepel v0.9.6. Major Python modules and versions are reported in the corresponding analytical subsections.

### 4.28 Data and code availability

The in-house sequencing study has been deposited in the NCBI Gene Expression Omnibus (GEO) under accession GSE346820 (https://www.ncbi.nlm.nih.gov/geo/query/acc.cgi?acc=GSE346820). Public access will be provided by publication in accordance with journal policy and applicable consent and institutional requirements. Revision-specific statistical tables and reproducible scripts are included as **Supplementary Data R1–R4** and Supplementary Code. Original WB images are supplied separately with a source-image index. The revision code repository is https://github.com/mznwjwn/NYC-melanoma-reproducibility. Public datasets are listed in the data-availability section below.

## 5 Conclusions

The NYC programme connects patient-derived CENPF enrichment and NSUN5 perturbation findings with melanoma expression states and immune features. Malignant-cell analyses nominate MLX for mechanistic investigation, and treatment-associated NYC changes motivate further clinical study. These observations support a testable biological framework, not a complete linear reader-mediated mechanism, proliferation-independent prognostic marker or validated drug-sensitivity predictor.

## Data Availability

The datasets presented in this study can be found in online repositories. The names of the repository/repositories and accession number(s) can be found in the article/**Supplementary Material**. The in-house sequencing data are deposited in the NCBI Gene Expression Omnibus (GEO) repository under accession number GSE346820 (https://www.ncbi.nlm.nih.gov/geo/query/acc.cgi?acc=GSE346820).
