# Supplementary material for: An m5C-associated NSUN5–YBX1–CENPF programme marks melanoma cell states and immune features
Source: Front Immunol. 2026 Oct 16;17:1952519. doi: 10.3389/fimmu.2026.1952519 (PMC13719537; doi:10.3389/fimmu.2026.1952519)
Supplement: Supplementary file 1 [file DataSheet1.pdf]

Supplementary Figure S2 | A

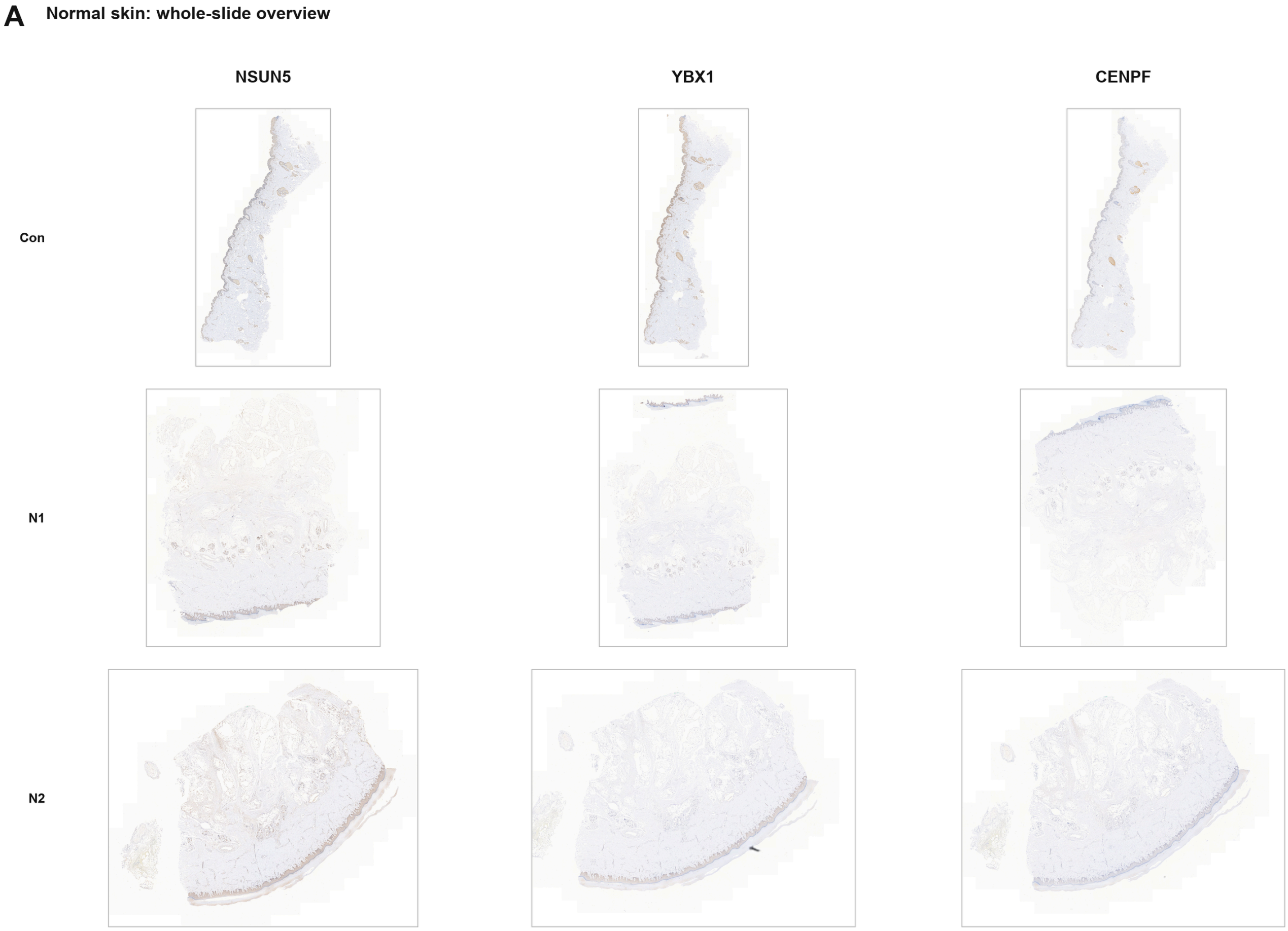

Normal-skin tissue immunohistochemistry source overview.

Supplementary Figure S2 | B

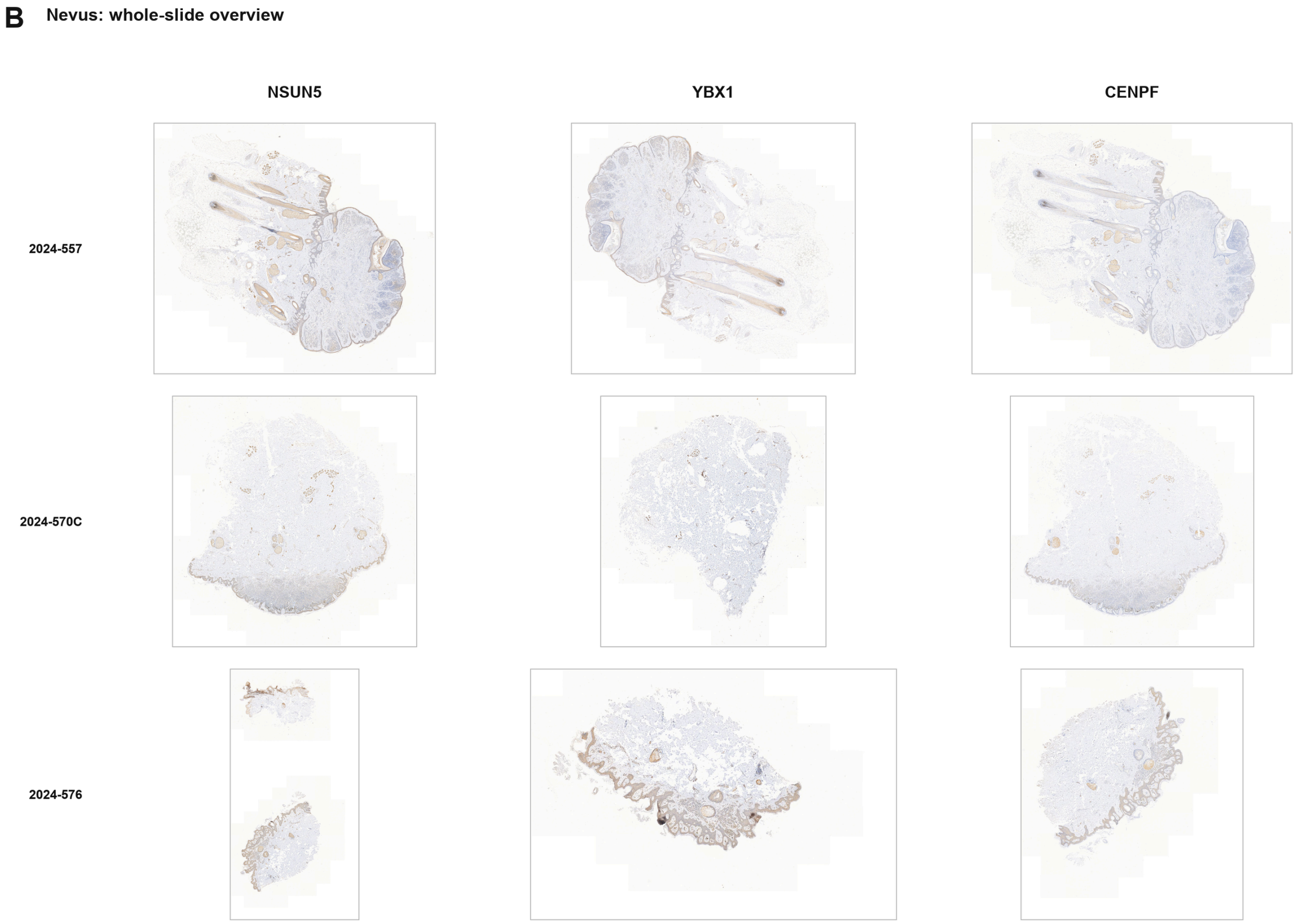

Naevus tissue immunohistochemistry source overview.

Supplementary Figure S2 | C

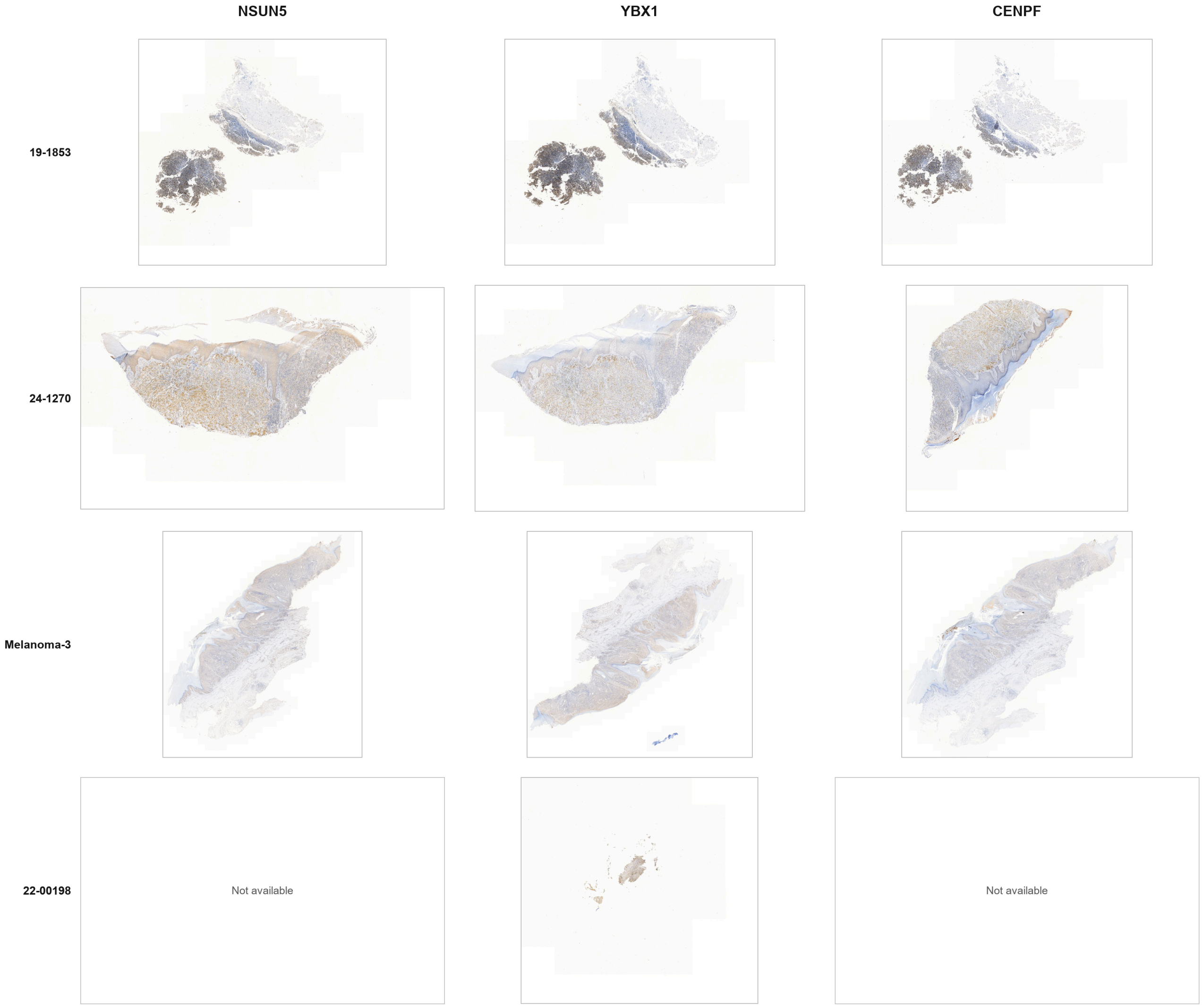

Case 22-00198 contains YBX1 only and is excluded from matched three-marker comparisons.

Melanoma tissue immunohistochemistry source overview.

Supplementary Figure S2 | D

D **22-00198: YBX1 only**

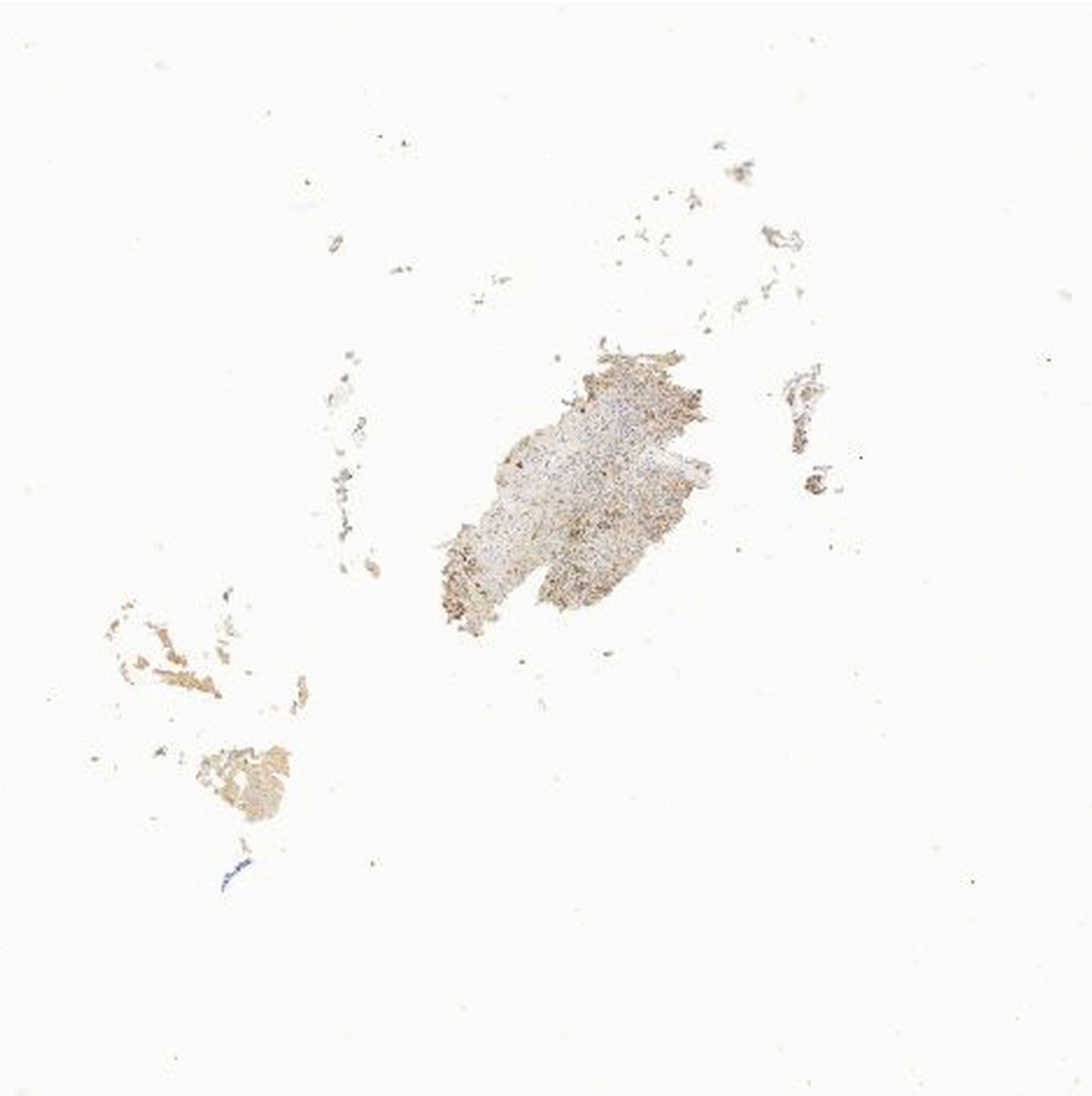

Retained enlarged YBX1 tissue field from the original supplementary figure.
