# Supplementary material for: An m5C-associated NSUN5–YBX1–CENPF programme marks melanoma cell states and immune features
Source: Front Immunol. 2026 Oct 16;17:1952519. doi: 10.3389/fimmu.2026.1952519 (PMC13719537; doi:10.3389/fimmu.2026.1952519)
Supplement: Supplementary file 2 [file DataSheet2.pdf]

Supplementary Figure S3 | A

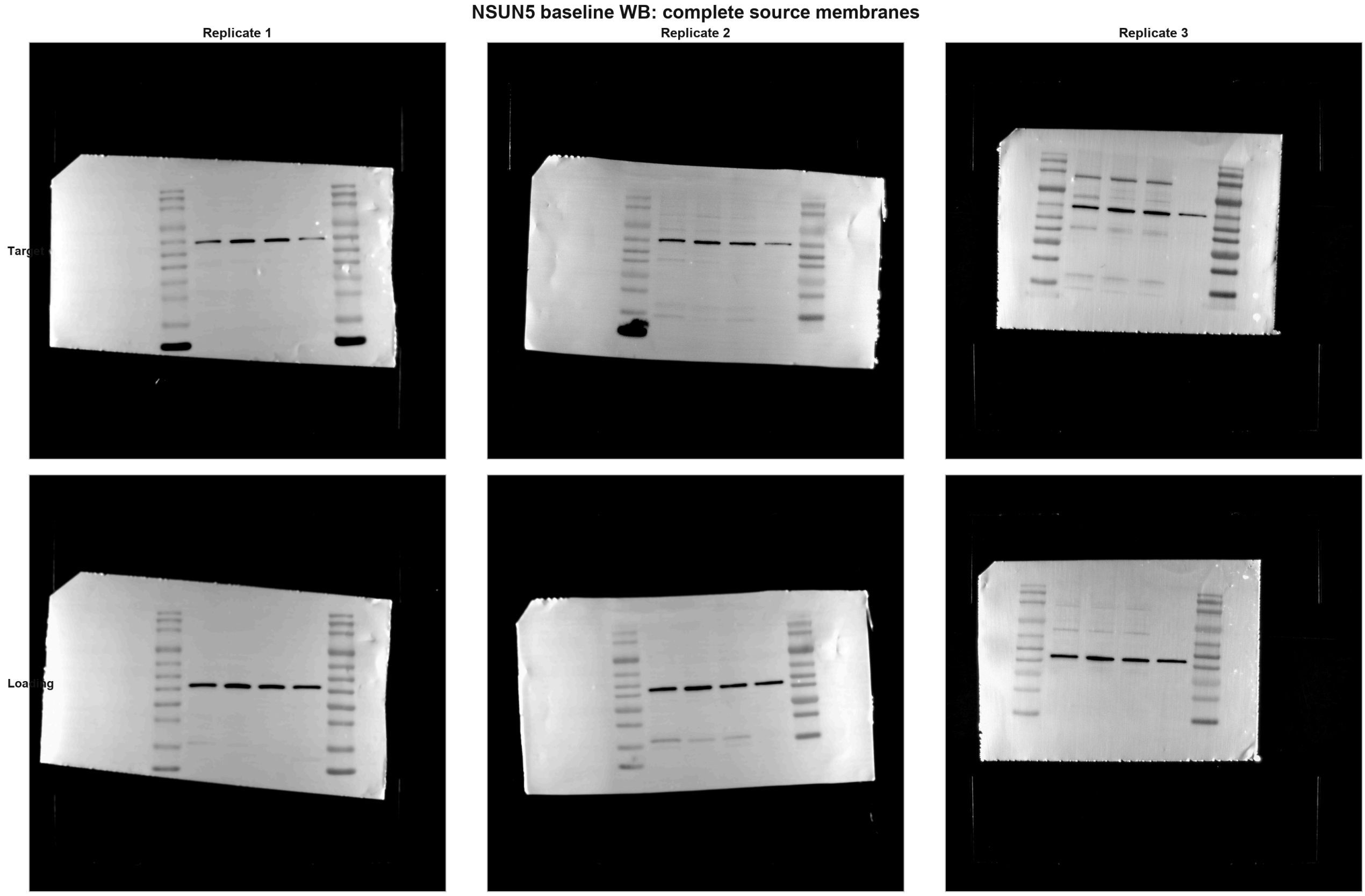

NSUN5 baseline western blots. Three replicates are shown with their corresponding loading-control images.

Supplementary Figure S3 | B

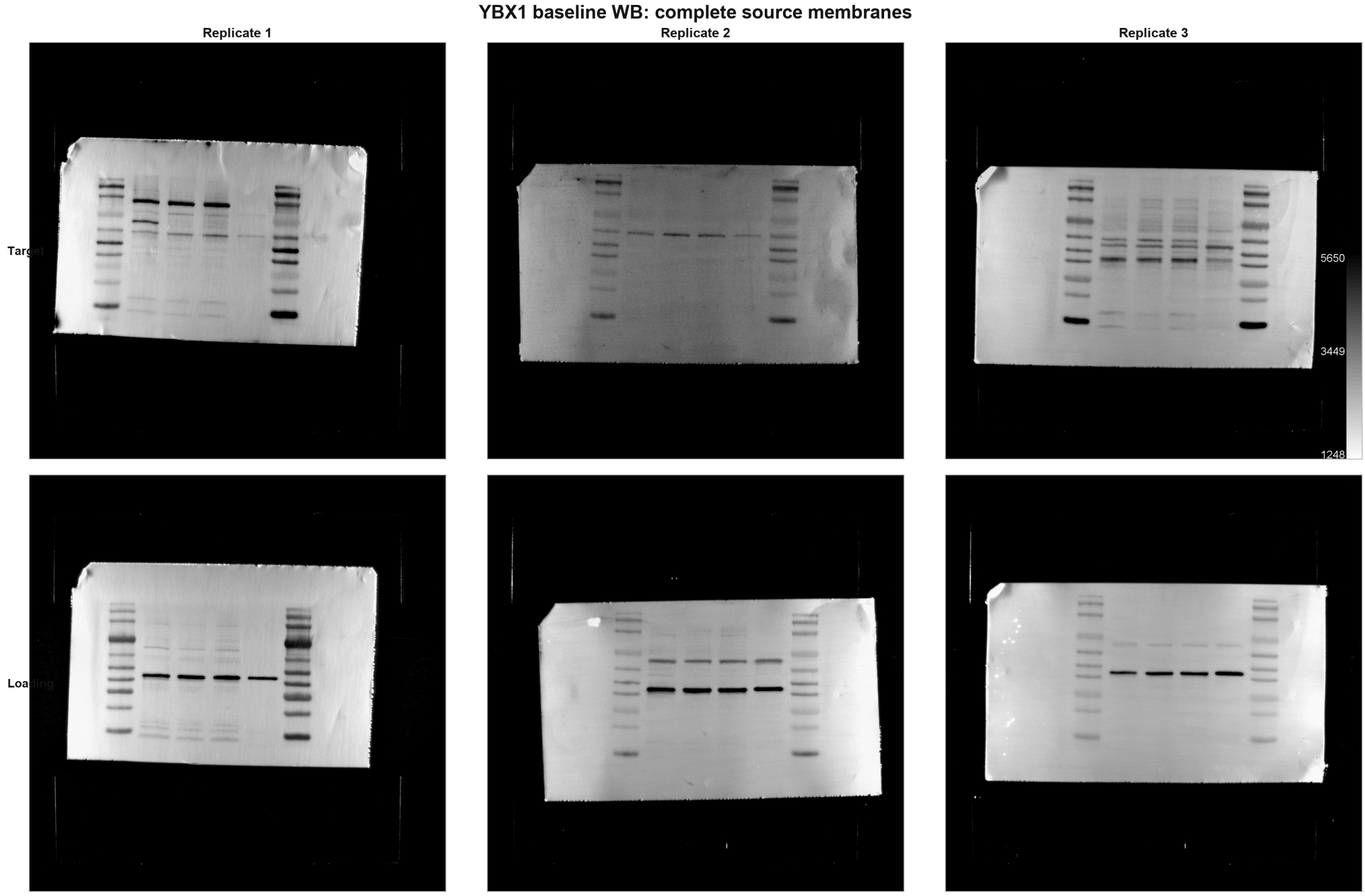

YBX1 baseline western blots. Three replicates are shown with their corresponding loading-control images.

Supplementary Figure S3 | C

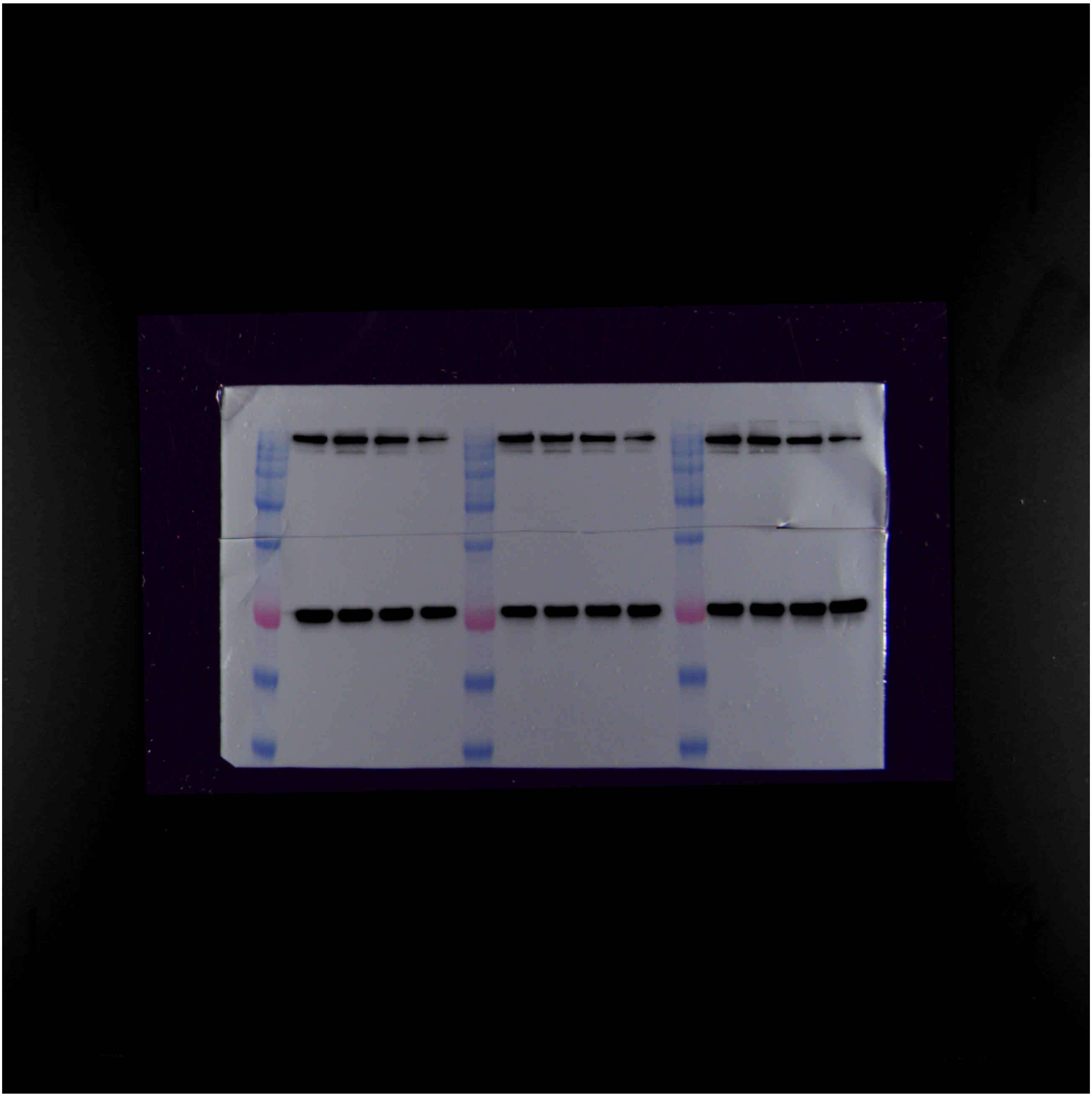

CENPF baseline: complete author-supplied frame, including the surrounding black border. The same membrane frame contains CENPF and HSP90 regions.
