# Supplementary material for: An m5C-associated NSUN5–YBX1–CENPF programme marks melanoma cell states and immune features
Source: Front Immunol. 2026 Oct 16;17:1952519. doi: 10.3389/fimmu.2026.1952519 (PMC13719537; doi:10.3389/fimmu.2026.1952519)
Supplement: Supplementary file 3 [file DataSheet3.pdf]

Supplementary Figure S4 | A

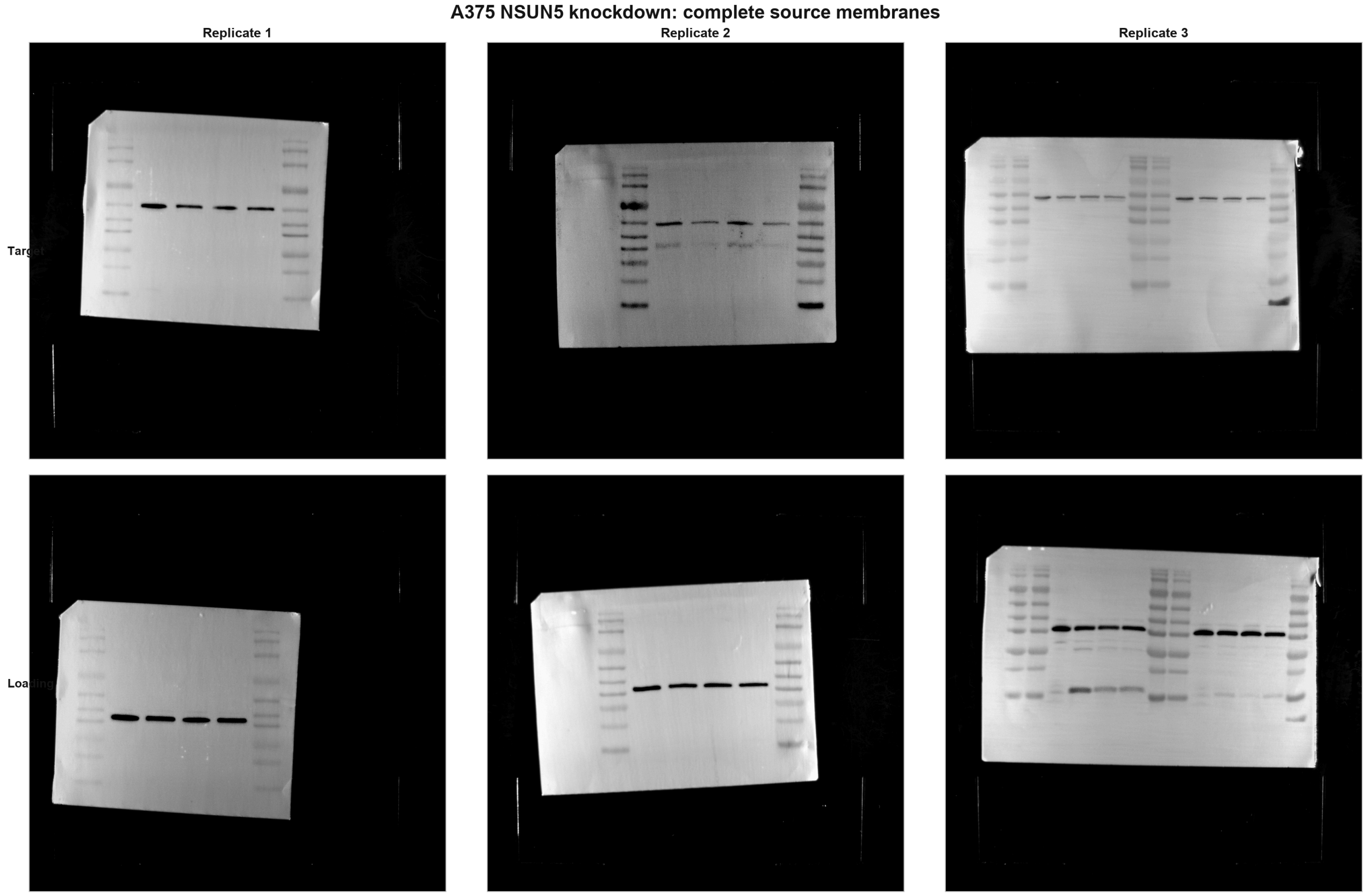

A375 NSUN5-knockdown source blots, including the corresponding loading-control images.

Supplementary Figure S4 | B

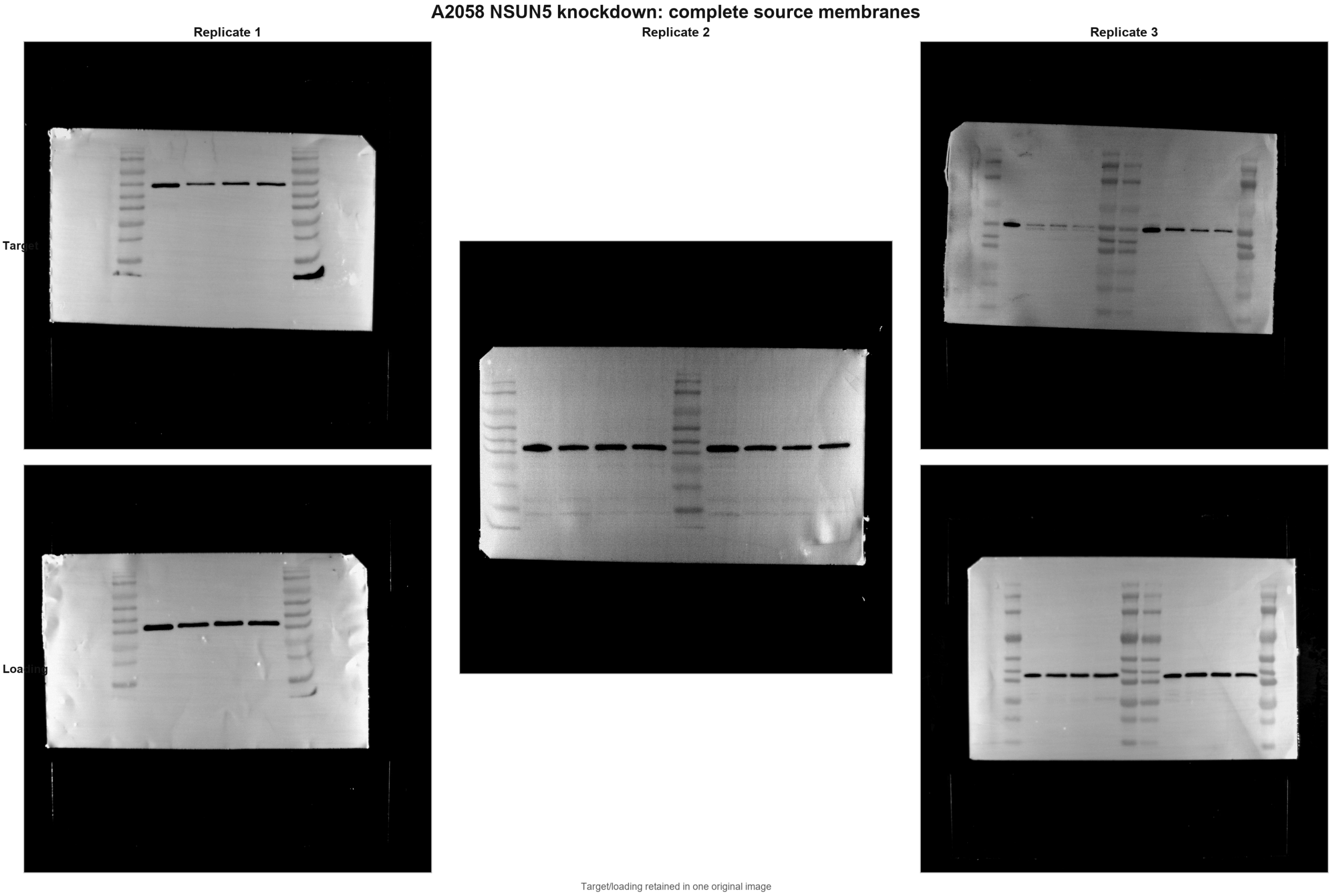

A2058 NSUN5-knockdown source blots, including the corresponding loading-control images.

Supplementary Figure S4 | C

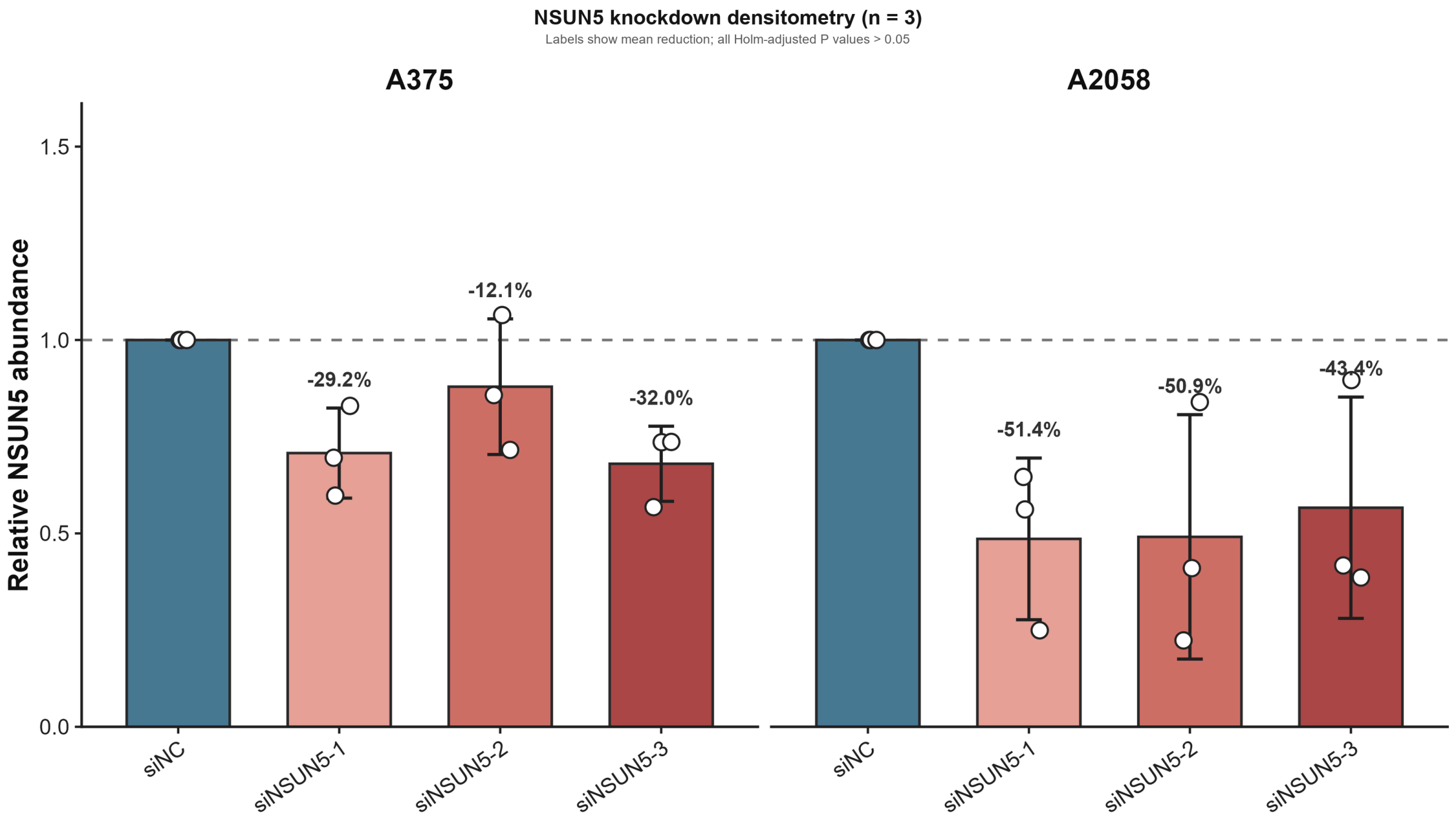

NSUN5-knockdown densitometry across the three siRNA constructs. Quantitative values and statistical definitions are unchanged.
