# Supplementary material for: An m5C-associated NSUN5–YBX1–CENPF programme marks melanoma cell states and immune features
Source: Front Immunol. 2026 Oct 16;17:1952519. doi: 10.3389/fimmu.2026.1952519 (PMC13719537; doi:10.3389/fimmu.2026.1952519)
Supplement: Supplementary file 4 [file DataSheet4.pdf]

Supplementary Figure S5 | A,B

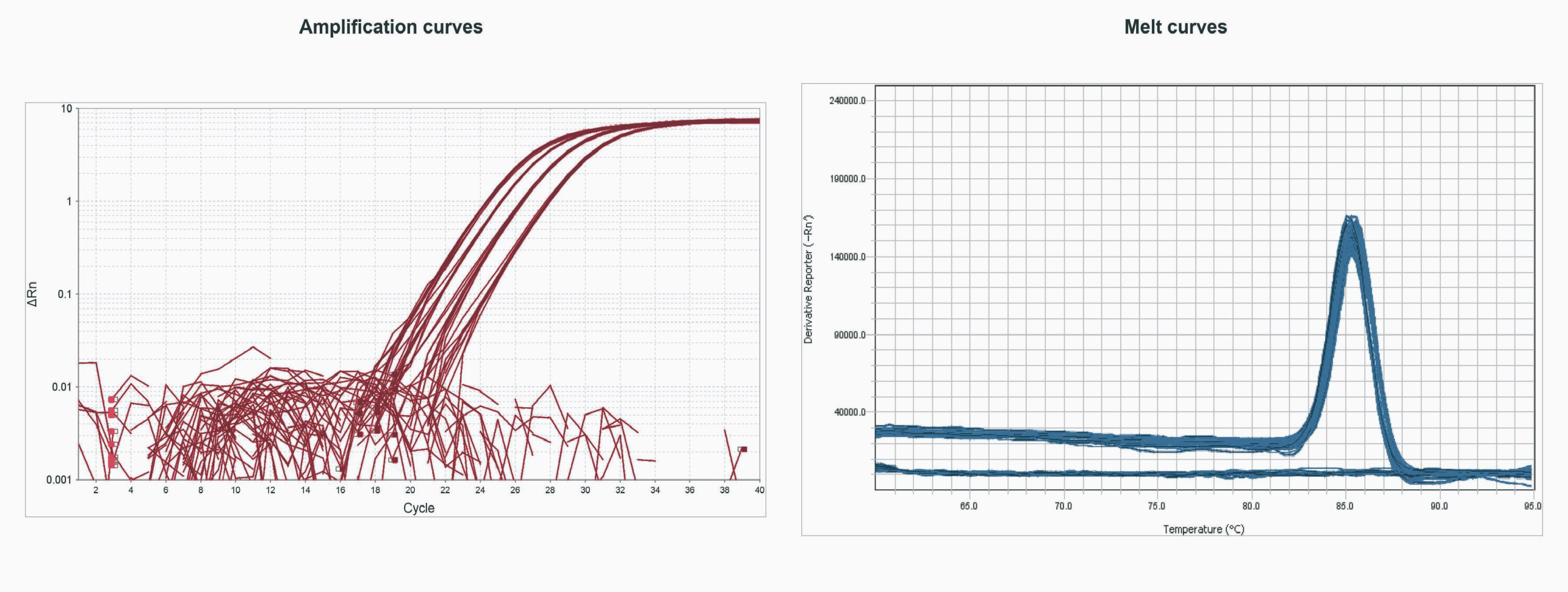

CENPF MeRIP-qPCR amplification (A) and melting (B) curves.

Supplementary Figure S5 | C

CENPF protein after NSUN5 knockdown: complete source image

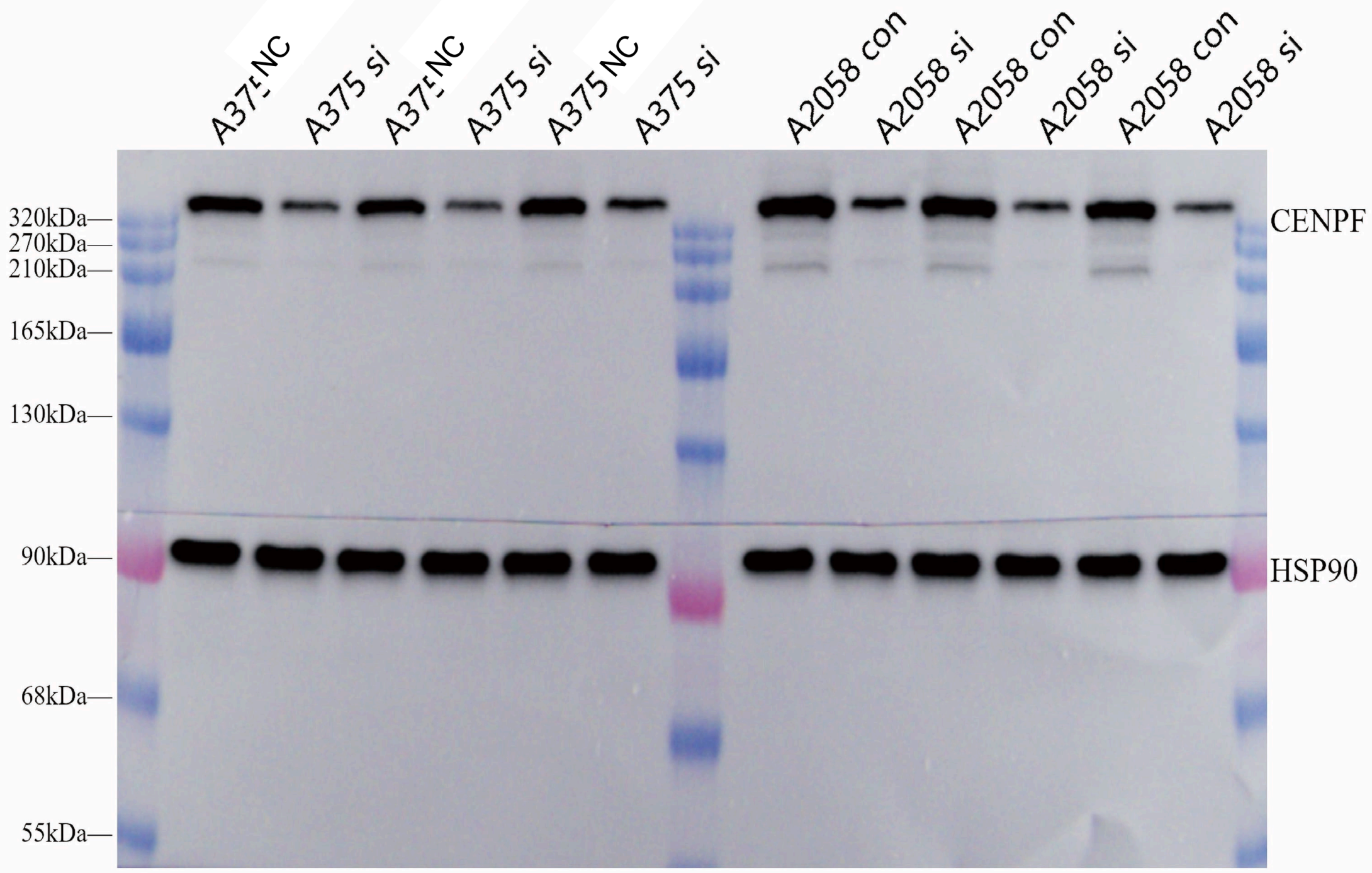

CENPF and HSP90 source image after NSUN5 knockdown in A375 and A2058 cells. The revised Fig. 3H retains the complete terminal lane in each displayed group.
