# Supplementary material for: An m5C-associated NSUN5–YBX1–CENPF programme marks melanoma cell states and immune features
Source: Front Immunol. 2026 Oct 16;17:1952519. doi: 10.3389/fimmu.2026.1952519 (PMC13719537; doi:10.3389/fimmu.2026.1952519)
Supplement: Supplementary file 5 [file DataSheet5.pdf]

Supplementary Figure S6 | A

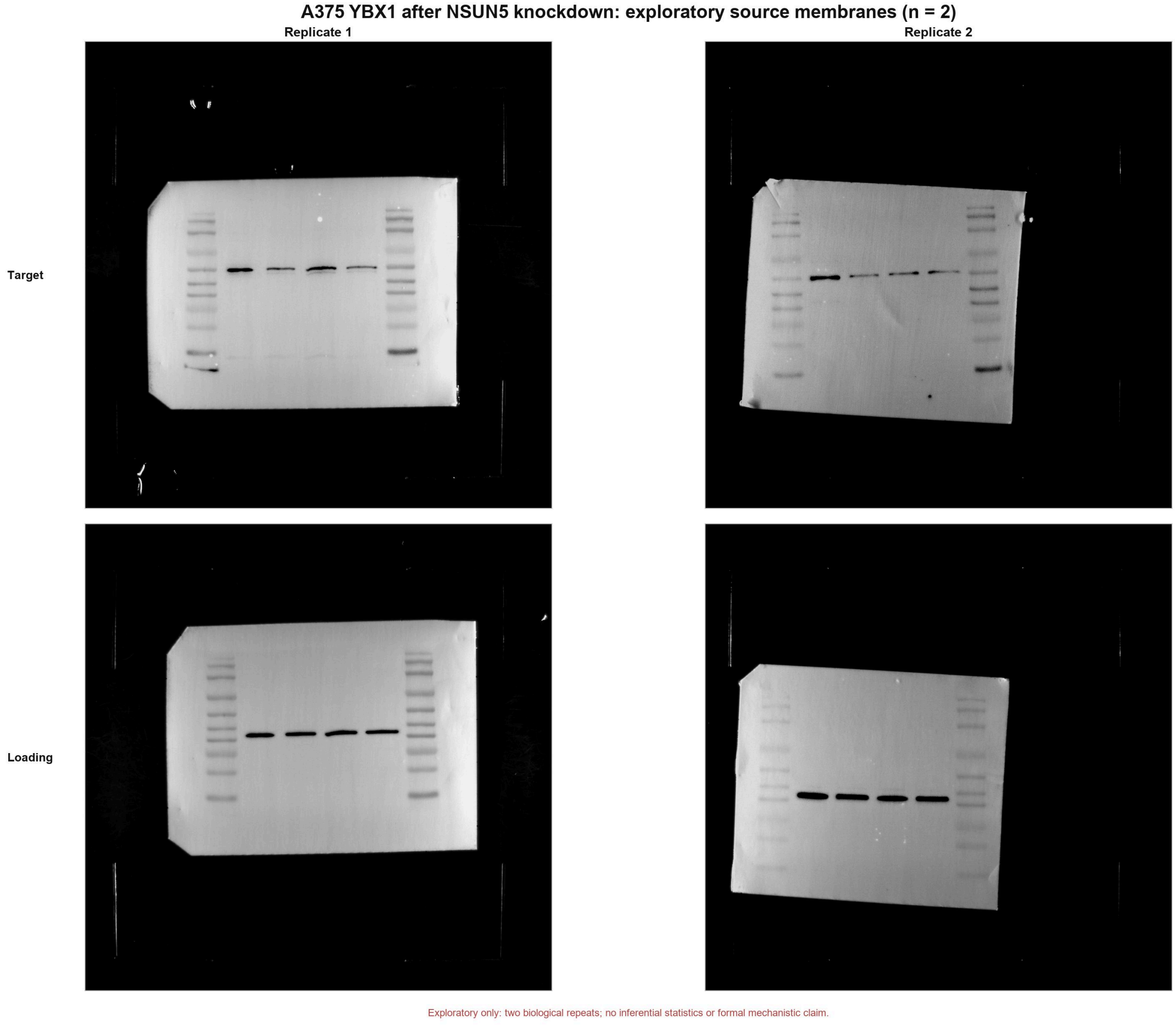

A375 YBX1 after NSUN5 knockdown: exploratory source membranes from two experiments.

Supplementary Figure S6 | B

A2058 YBX1/NSUN5 source exposures after NSUN5 knockdown (n = 2)

Replicate 1

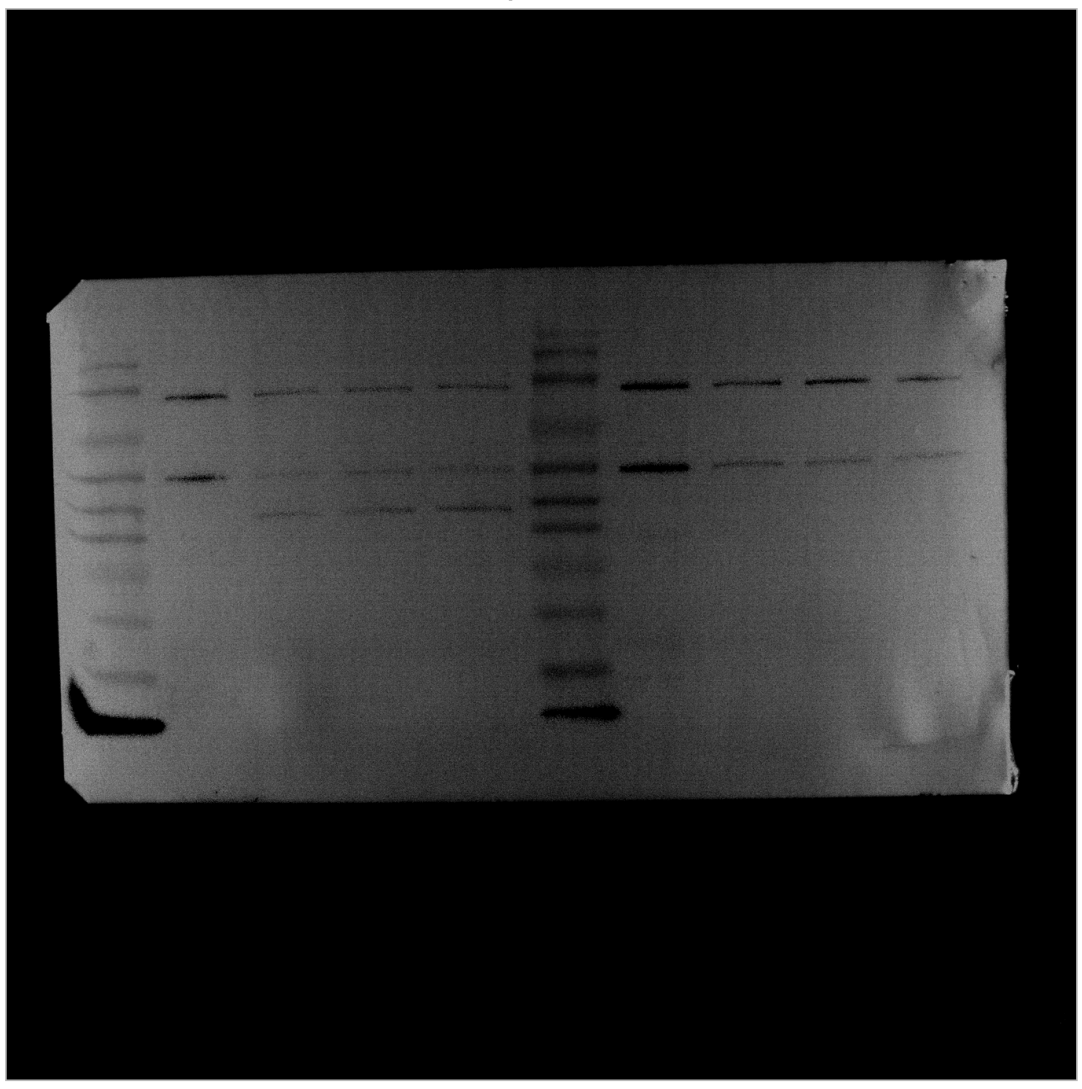

Replicate 2

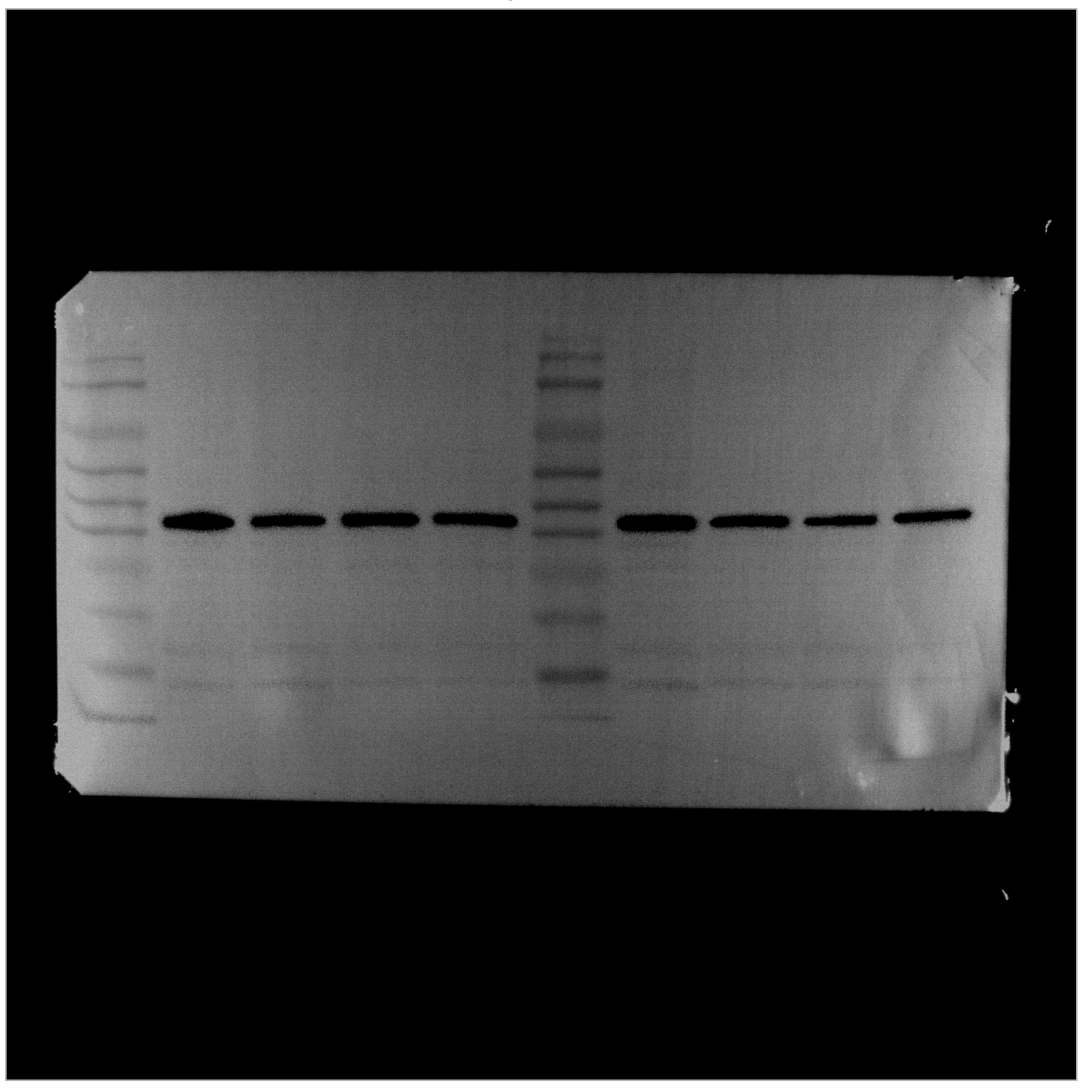

Exploratory only: two source exposures; no densitometric statistics or formal mechanistic claim.

A2058 YBX1/NSUN5 source exposures after NSUN5 knockdown: two experiments. These observations do not establish a YBX1-mediated effect on CENPF.
