# Supplementary material for: An m5C-associated NSUN5–YBX1–CENPF programme marks melanoma cell states and immune features
Source: Front Immunol. 2026 Oct 16;17:1952519. doi: 10.3389/fimmu.2026.1952519 (PMC13719537; doi:10.3389/fimmu.2026.1952519)
Supplement: Supplementary file 6 [file Image1.pdf]

Supplementary Figure S1 | Peak calls and clinical associations

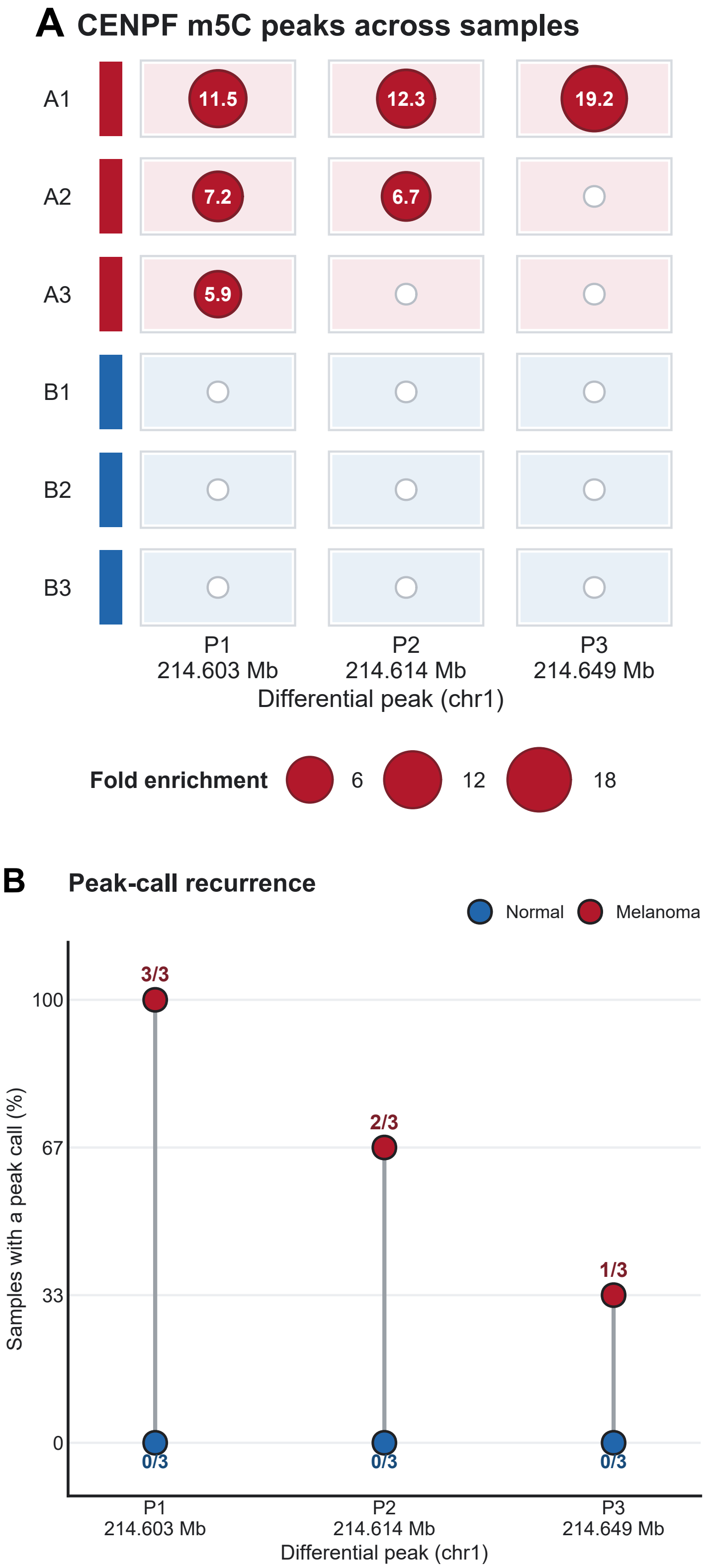

A, CENPF sample-level peak calls. B, Recurrence of the nominated CENPF regions across the sequenced samples. These descriptive peak calls do not replace the Input-adjusted interaction analysis in Fig. 2C.

Supplementary Figure S1 | C

id	HR	HR.95L	HR.95H	pvalue	Method
NSUN5	1.170	0.947	1.444	0.145	UniCox−TCGA
**Age**	**1.915**	**1.428**	**2.567**	**<0.001**	
Gender	1.149	0.861	1.534	0.345	
**Stage**	**1.403**	**1.174**	**1.677**	**<0.001**	
**T**	**1.465**	**1.272**	**1.687**	**<0.001**	
**N**	**1.360**	**1.189**	**1.556**	**<0.001**	
**M**	**1.896**	**1.029**	**3.496**	**0.04**	
NSUN5	1.154	0.912	1.461	0.233	MultiCox−TCGA
Age	1.353	0.961	1.905	0.083	
Gender	0.996	0.713	1.391	0.981	
Stage	0.790	0.567	1.100	0.162	
**T**	**1.587**	**1.341**	**1.879**	**<0.001**	
**N**	**1.645**	**1.314**	**2.059**	**<0.001**	
**M**	**2.559**	**1.069**	**6.127**	**0.035**	
**NSUN5**	**1.931**	**1.143**	**3.261**	**0.014**	UniCox−GSE19234
Age	1.197	0.522	2.746	0.67	
Gender	0.766	0.335	1.750	0.526	
**Stage**	**4.037**	**1.432**	**11.376**	**0.008**	
**NSUN5**	**1.708**	**1.022**	**2.854**	**0.041**	MultiCox−GSE19234
Age	1.113	0.461	2.687	0.811	
Gender	0.848	0.363	1.980	0.703	
**Stage**	**3.085**	**1.048**	**9.079**	**0.041**	

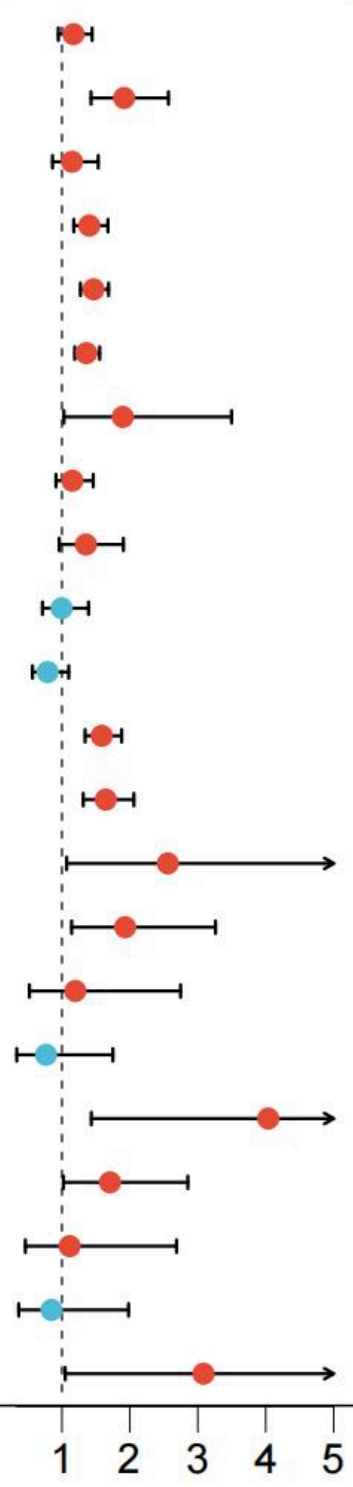

C, Archived cross-cohort Cox estimates for NSUN5. The plotted cohort, covariate, hazard-ratio and confidence-interval values are retained from the source figure.

Supplementary Figure S1 | D

id	HR	HR.95L	HR.95H	pvalue	Method
YBX1	1.321	1.009	1.730	0.043	UniCox−TCGA
Age	1.915	1.428	2.567	<0.001	
Gender	1.149	0.861	1.534	0.345	
Stage	1.403	1.174	1.677	<0.001	
T	1.465	1.272	1.687	<0.001	
N	1.360	1.189	1.556	<0.001	
M	1.896	1.029	3.496	0.04	
YBX1	1.452	1.090	1.935	0.011	MultiCox−TCGA
Age	1.293	0.916	1.825	0.143	
Gender	0.994	0.712	1.387	0.97	
Stage	0.795	0.573	1.105	0.172	
T	1.574	1.328	1.864	<0.001	
N	1.658	1.325	2.074	<0.001	
M	2.381	0.994	5.704	0.052	
YBX1	2.923	1.675	5.099	<0.001	UniCox−GSE98394
Gender	1.583	0.558	4.495	0.388	
Stage	6.929	2.380	20.176	<0.001	
T	4.167	1.944	8.934	<0.001	
N	3.072	1.691	5.581	<0.001	
YBX1	3.924	1.131	13.615	0.031	MultiCox−GSE98394
Gender	1.535	0.359	6.565	0.564	
Stage	0.637	0.110	3.684	0.615	
T	2.227	0.660	7.516	0.197	
N	6.278	1.254	31.420	0.025	

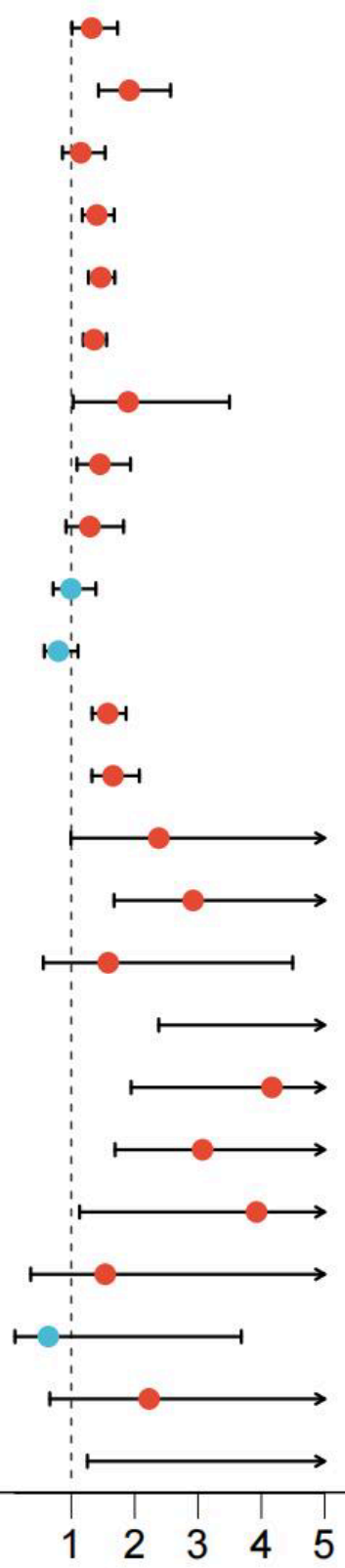

D, Archived cross-cohort Cox estimates for YBX1. The plotted cohort, covariate, hazard-ratio and confidence-interval values are retained from the source figure.

Supplementary Figure S1 | E

id	HR	HR.95L	HR.95H	pvalue	Method
CENPF	1.115	0.971	1.279	0.122	UniCox−TCGA
**Age**	**1.915**	**1.428**	**2.567**	**<0.001**	
Gender	1.149	0.861	1.534	0.345	
**Stage**	**1.403**	**1.174**	**1.677**	**<0.001**	
**T**	**1.465**	**1.272**	**1.687**	**<0.001**	
**N**	**1.360**	**1.189**	**1.556**	**<0.001**	
**M**	**1.896**	**1.029**	**3.496**	**0.04**	
CENPF	1.133	0.952	1.348	0.16	MultiCox−TCGA
Age	1.291	0.912	1.828	0.15	
Gender	0.970	0.693	1.358	0.861	
Stage	0.796	0.572	1.108	0.176	
**T**	**1.604**	**1.352**	**1.902**	**<0.001**	
**N**	**1.640**	**1.311**	**2.052**	**<0.001**	
**M**	**2.419**	**1.011**	**5.788**	**0.047**	
**CENPF**	**1.774**	**1.163**	**2.703**	**0.008**	UniCox−GSE19234
Age	1.197	0.522	2.746	0.67	
Gender	0.766	0.335	1.750	0.526	
**Stage**	**4.037**	**1.432**	**11.376**	**0.008**	
**CENPF**	**1.640**	**1.044**	**2.575**	**0.032**	MultiCox−GSE19234
Age	1.204	0.513	2.827	0.67	
Gender	0.924	0.395	2.162	0.855	
Stage	2.843	0.969	8.338	0.057	

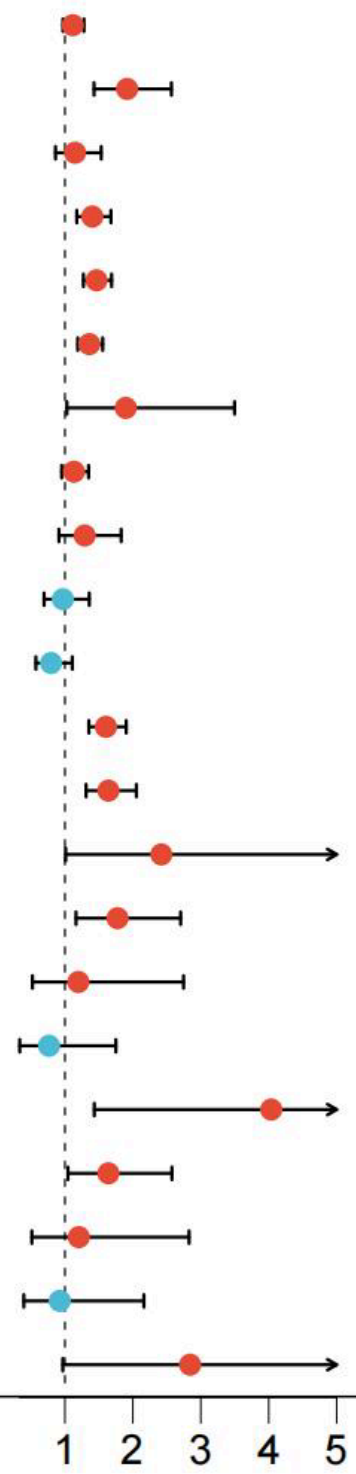

E, Archived cross-cohort Cox estimates for CENPF. The plotted cohort, covariate, hazard-ratio and confidence-interval values are retained from the source figure.
