# Supplementary material for: An m5C-associated NSUN5–YBX1–CENPF programme marks melanoma cell states and immune features
Source: Front Immunol. 2026 Oct 16;17:1952519. doi: 10.3389/fimmu.2026.1952519 (PMC13719537; doi:10.3389/fimmu.2026.1952519)
Supplement: Supplementary file 7 [file Table1.docx]

Supplementary Western blot source images

Source-image collection accompanying Figure 3 and Supplementary Figures S3-S6.

This supplement presents 49 source-file previews, arranged by the corresponding experiment and figure panel. Each preview is identified by the filename of its accompanying TIFF or JPEG file. File numbers identify source files, not independent biological replicates.

Previews are reproduced from the existing source-image index without additional image processing. They are scaled proportionally for display. The separate source files retain their original resolution and should be consulted for detailed image assessment.

# Panel and replicate crosswalk

Use the numbered entries below to locate the exact source filenames in the existing image index. Target and control entries are linked by experiment and replicate. A source-file number is not a biological replicate. The 49 index entries correspond to 50 archived image files because the later complete CENPF baseline JPG is included alongside the original TIFF exports.

The two upload ZIP parts together contain the complete WB_source_images.zip collection, with the same image bytes. WB_panel_mapping.csv and WB_file_manifest.csv provide searchable English-language mappings. Source TIFF/JPEG files, not the scaled Word previews, are the resolution-preserving records.

## NSUN5 baseline

Figure 3B and Supplementary Figure S3A

Sample lanes 1-4: A375, A2058, 451Lu, HEMa; marker lanes excluded.

| **Replicate** | **Target entry** | **Control entry** | **Displayed use** |
| --- | --- | --- | --- |
| R1 | 04 | 01 | Figure 3B representative; S3A R1 |
| R2 | 10 | 07 | S3A R2 |
| R3 | 19 | 16 | S3A R3; 13 is an alternate control exposure |

## YBX1 baseline

Figure 3B and Supplementary Figure S3B

Sample lanes 1-4: A375, A2058, 451Lu, HEMa; marker lanes excluded.

| **Replicate** | **Target entry** | **Control entry** | **Displayed use** |
| --- | --- | --- | --- |
| R1 | 22 | 23 | Figure 3B representative; S3B R1 |
| R2 | 24 | 25 | S3B R2 |
| R3 | 26 | 27 | S3B R3 |

## CENPF baseline

Figure 3B and Supplementary Figure S3C

Three consecutive four-sample groups are R1, R2 and R3. Within each group: A375, A2058, 451Lu, HEMa. CENPF is the upper region; HSP90 is the lower region. Figure 3B shows R1.

| **Replicate** | **Target entry** | **Control entry** | **Displayed use** |
| --- | --- | --- | --- |
| R1-R3 | 28 | 29 | Both previews show CENPF_baseline_full_frame.jpg; not independent frames |

## A375 NSUN5 knockdown screening

Figure 3F and Supplementary Figure S4A

Sample lanes 1-4: siNC, siN5-1, siN5-2, siN5-3; marker lanes excluded.

| **Replicate** | **Target entry** | **Control entry** | **Displayed use** |
| --- | --- | --- | --- |
| R1 | 30 | 31 | Figure 3F representative; S4A R1 |
| R2 | 32 | 33 | S4A R2 |
| R3 | 34 | 35 | S4A R3 |

## A2058 NSUN5 knockdown screening

Figure 3F and Supplementary Figure S4B

Sample lanes 1-4: siNC, siN5-1, siN5-2, siN5-3; marker lanes excluded.

| **Replicate** | **Target entry** | **Control entry** | **Displayed use** |
| --- | --- | --- | --- |
| R1 | 37 | 36 | Figure 3F representative; S4B R1 |
| R2 | 41 | 41 | Combined exposure; 40 is the accompanying marker channel |
| R3 | 38 | 39 | S4B R3 |

## CENPF after NSUN5 knockdown

Figure 3H and Supplementary Figure S5C

A375 is followed by A2058. Each cell-line block contains R1 siNC/siNSUN5, R2 siNC/siNSUN5 and R3 siNC/siNSUN5. Corresponding HSP90 lanes are vertically aligned with the target lanes.

| **Replicate** | **Target entry** | **Control entry** | **Displayed use** |
| --- | --- | --- | --- |
| R1-R3 per cell line | 42 | 43 | Same combined CENPF/HSP90 frame under two supplied filenames |

## A375 YBX1 exploratory blots

Supplementary Figure S6A

Sample lanes 1-4: siNC, siN5-1, siN5-2, siN5-3; marker lanes excluded.

| **Replicate** | **Target entry** | **Control entry** | **Displayed use** |
| --- | --- | --- | --- |
| R1 | 45 | 44 | Two exploratory experiments only |
| R2 | 46 | 47 | Entry 47 is byte-identical to entry 31; not a new independent control file |

## A2058 exploratory source exposures

Supplementary Figure S6B

Sample lanes 1-4: siNC, siN5-1, siN5-2, siN5-3; marker lanes excluded.

| **Replicate** | **Target entry** | **Control entry** | **Displayed use** |
| --- | --- | --- | --- |
| Source exposure 1 | 48 | 48 | Combined source exposure; no new band assignment or quantification |
| Source exposure 2 | 49 | 49 | Same source file as entry 41, reused for documentation, not independent evidence |

## Duplicate source records

The supplied archive contains duplicate exports and reused documentation records. Entries 28/29 and 42/43 respectively contain target and loading-control regions within the same image, rather than two independent membranes. Entries 15/18 are identical marker-channel files. Entries 31/47 share the same supplied control file, and entries 41/49 share the same supplied combined exposure. These identities are explicitly listed in the manifest and are not counted as additional experiments. The S6 observations remain exploratory; no new mechanistic or quantitative conclusion is drawn from the reused files.

CENPF baseline entries 28 and 29 display the complete author-supplied JPG with its black border retained. The older TIFF exports are preserved in the archive. No image content, contrast, band shape or crop has been changed in this documentation update.

# Image index

| **Figure panel** | **Experiment** | **Images** |
| --- | --- | --- |
| Figure 3B; S3A | NSUN5 baseline expression in A375, A2058, 451Lu and HEMa | 01-21 |
| Figure 3B; S3B | YBX1 baseline expression in A375, A2058, 451Lu and HEMa | 22-27 |
| Figure 3B; S3C | CENPF baseline expression in A375, A2058, 451Lu and HEMa | 28-29 |
| Figure 3F; S4A | NSUN5 knockdown screening in A375 cells | 30-35 |
| Figure 3F; S4B | NSUN5 knockdown screening in A2058 cells | 36-41 |
| Figure 3H and Supplementary Figure S5C | CENPF protein abundance after NSUN5 knockdown | 42-43 |
| Supplementary Figure S6A | Exploratory YBX1 source blots after NSUN5 knockdown in A375 cells | 44-47 |
| Supplementary Figure S6B | Exploratory YBX1 source blots after NSUN5 knockdown in A2058 cells | 48-49 |

# Source image 01

**Figure 3B**

NSUN5 baseline expression in A375, A2058, 451Lu and HEMa

**Source file:** 01_Figure3B_NSUN5_baseline_01.tif

**File description:** loading-control or combined exposure

Source image 01. Preview of the accompanying source file; image content and aspect ratio are preserved.

# Source image 02

**Figure 3B**

NSUN5 baseline expression in A375, A2058, 451Lu and HEMa

**Source file:** 01_Figure3B_NSUN5_baseline_02.tif

**File description:** loading-control or combined exposure

Source image 02. Preview of the accompanying source file; image content and aspect ratio are preserved.

# Source image 03

**Figure 3B**

NSUN5 baseline expression in A375, A2058, 451Lu and HEMa

**Source file:** 01_Figure3B_NSUN5_baseline_03.tif

**File description:** loading-control or combined exposure

Source image 03. Preview of the accompanying source file; image content and aspect ratio are preserved.

# Source image 04

**Figure 3B**

NSUN5 baseline expression in A375, A2058, 451Lu and HEMa

**Source file:** 01_Figure3B_NSUN5_baseline_04.tif

**File description:** NSUN5 target or combined exposure

Source image 04. Preview of the accompanying source file; image content and aspect ratio are preserved.

# Source image 05

**Figure 3B**

NSUN5 baseline expression in A375, A2058, 451Lu and HEMa

**Source file:** 01_Figure3B_NSUN5_baseline_05.tif

**File description:** NSUN5 target or combined exposure

Source image 05. Preview of the accompanying source file; image content and aspect ratio are preserved.

# Source image 06

**Figure 3B**

NSUN5 baseline expression in A375, A2058, 451Lu and HEMa

**Source file:** 01_Figure3B_NSUN5_baseline_06.tif

**File description:** NSUN5 target or combined exposure

Source image 06. Preview of the accompanying source file; image content and aspect ratio are preserved.

# Source image 07

**Figure 3B**

NSUN5 baseline expression in A375, A2058, 451Lu and HEMa

**Source file:** 01_Figure3B_NSUN5_baseline_07.tif

**File description:** loading-control or combined exposure

Source image 07. Preview of the accompanying source file; image content and aspect ratio are preserved.

# Source image 08

**Figure 3B**

NSUN5 baseline expression in A375, A2058, 451Lu and HEMa

**Source file:** 01_Figure3B_NSUN5_baseline_08.tif

**File description:** loading-control or combined exposure

Source image 08. Preview of the accompanying source file; image content and aspect ratio are preserved.

# Source image 09

**Figure 3B**

NSUN5 baseline expression in A375, A2058, 451Lu and HEMa

**Source file:** 01_Figure3B_NSUN5_baseline_09.tif

**File description:** loading-control or combined exposure

Source image 09. Preview of the accompanying source file; image content and aspect ratio are preserved.

# Source image 10

**Figure 3B**

NSUN5 baseline expression in A375, A2058, 451Lu and HEMa

**Source file:** 01_Figure3B_NSUN5_baseline_10.tif

**File description:** NSUN5 target or combined exposure

Source image 10. Preview of the accompanying source file; image content and aspect ratio are preserved.

# Source image 11

**Figure 3B**

NSUN5 baseline expression in A375, A2058, 451Lu and HEMa

**Source file:** 01_Figure3B_NSUN5_baseline_11.tif

**File description:** NSUN5 target or combined exposure

Source image 11. Preview of the accompanying source file; image content and aspect ratio are preserved.

# Source image 12

**Figure 3B**

NSUN5 baseline expression in A375, A2058, 451Lu and HEMa

**Source file:** 01_Figure3B_NSUN5_baseline_12.tif

**File description:** NSUN5 target or combined exposure

Source image 12. Preview of the accompanying source file; image content and aspect ratio are preserved.

# Source image 13

**Figure 3B**

NSUN5 baseline expression in A375, A2058, 451Lu and HEMa

**Source file:** 01_Figure3B_NSUN5_baseline_13.tif

**File description:** loading-control or combined exposure

Source image 13. Preview of the accompanying source file; image content and aspect ratio are preserved.

# Source image 14

**Figure 3B**

NSUN5 baseline expression in A375, A2058, 451Lu and HEMa

**Source file:** 01_Figure3B_NSUN5_baseline_14.tif

**File description:** loading-control or combined exposure

Source image 14. Preview of the accompanying source file; image content and aspect ratio are preserved.

# Source image 15

**Figure 3B**

NSUN5 baseline expression in A375, A2058, 451Lu and HEMa

**Source file:** 01_Figure3B_NSUN5_baseline_15.tif

**File description:** loading-control or combined exposure

Source image 15. Preview of the accompanying source file; image content and aspect ratio are preserved.

# Source image 16

**Figure 3B**

NSUN5 baseline expression in A375, A2058, 451Lu and HEMa

**Source file:** 01_Figure3B_NSUN5_baseline_16.tif

**File description:** loading-control or combined exposure

Source image 16. Preview of the accompanying source file; image content and aspect ratio are preserved.

# Source image 17

**Figure 3B**

NSUN5 baseline expression in A375, A2058, 451Lu and HEMa

**Source file:** 01_Figure3B_NSUN5_baseline_17.tif

**File description:** loading-control or combined exposure

Source image 17. Preview of the accompanying source file; image content and aspect ratio are preserved.

# Source image 18

**Figure 3B**

NSUN5 baseline expression in A375, A2058, 451Lu and HEMa

**Source file:** 01_Figure3B_NSUN5_baseline_18.tif

**File description:** loading-control or combined exposure

Source image 18. Preview of the accompanying source file; image content and aspect ratio are preserved.

# Source image 19

**Figure 3B**

NSUN5 baseline expression in A375, A2058, 451Lu and HEMa

**Source file:** 01_Figure3B_NSUN5_baseline_19.tif

**File description:** NSUN5 target or combined exposure

Source image 19. Preview of the accompanying source file; image content and aspect ratio are preserved.

# Source image 20

**Figure 3B**

NSUN5 baseline expression in A375, A2058, 451Lu and HEMa

**Source file:** 01_Figure3B_NSUN5_baseline_20.tif

**File description:** NSUN5 target or combined exposure

Source image 20. Preview of the accompanying source file; image content and aspect ratio are preserved.

# Source image 21

**Figure 3B**

NSUN5 baseline expression in A375, A2058, 451Lu and HEMa

**Source file:** 01_Figure3B_NSUN5_baseline_21.tif

**File description:** NSUN5 target or combined exposure

Source image 21. Preview of the accompanying source file; image content and aspect ratio are preserved.

# Source image 22

**Figure 3B**

YBX1 baseline expression in A375, A2058, 451Lu and HEMa

**Source file:** 02_Figure3B_YBX1_baseline_01.tif

**File description:** loading-control or combined exposure

Source image 22. Preview of the accompanying source file; image content and aspect ratio are preserved.

# Source image 23

**Figure 3B**

YBX1 baseline expression in A375, A2058, 451Lu and HEMa

**Source file:** 02_Figure3B_YBX1_baseline_02.tif

**File description:** loading-control or combined exposure

Source image 23. Preview of the accompanying source file; image content and aspect ratio are preserved.

# Source image 24

**Figure 3B**

YBX1 baseline expression in A375, A2058, 451Lu and HEMa

**Source file:** 02_Figure3B_YBX1_baseline_03.jpg

**File description:** loading-control or combined exposure

Source image 24. Preview of the accompanying source file; image content and aspect ratio are preserved.

# Source image 25

**Figure 3B**

YBX1 baseline expression in A375, A2058, 451Lu and HEMa

**Source file:** 02_Figure3B_YBX1_baseline_04.tif

**File description:** loading-control or combined exposure

Source image 25. Preview of the accompanying source file; image content and aspect ratio are preserved.

# Source image 26

**Figure 3B**

YBX1 baseline expression in A375, A2058, 451Lu and HEMa

**Source file:** 02_Figure3B_YBX1_baseline_05.tif

**File description:** YBX1 target or combined exposure

Source image 26. Preview of the accompanying source file; image content and aspect ratio are preserved.

# Source image 27

**Figure 3B**

YBX1 baseline expression in A375, A2058, 451Lu and HEMa

**Source file:** 02_Figure3B_YBX1_baseline_06.tif

**File description:** loading-control or combined exposure

Source image 27. Preview of the accompanying source file; image content and aspect ratio are preserved.

# Source image 28

**Figure 3B**

CENPF baseline expression in A375, A2058, 451Lu and HEMa

**Source file:** CENPF_baseline_full_frame.jpg

**File description:** CENPF target

Source image 28. Preview of the accompanying source file; image content and aspect ratio are preserved. Images 28 and 29 show the same full-frame image, indexed for CENPF and HSP90; they are not separate experiments.

# Source image 29

**Figure 3B**

CENPF baseline expression in A375, A2058, 451Lu and HEMa

**Source file:** CENPF_baseline_full_frame.jpg

**File description:** HSP90 loading control

Source image 29. Preview of the accompanying source file; image content and aspect ratio are preserved. Images 28 and 29 show the same full-frame image, indexed for CENPF and HSP90; they are not separate experiments.

# Source image 30

**Figure 3F**

NSUN5 knockdown screening in A375 cells

**Source file:** 04_Figure3F_A375_NSUN5_knockdown_01.tif

**File description:** NSUN5 target or combined exposure

Source image 30. Preview of the accompanying source file; image content and aspect ratio are preserved.

# Source image 31

**Figure 3F**

NSUN5 knockdown screening in A375 cells

**Source file:** 04_Figure3F_A375_NSUN5_knockdown_02.tif

**File description:** loading-control or combined exposure

Source image 31. Preview of the accompanying source file; image content and aspect ratio are preserved.

# Source image 32

**Figure 3F**

NSUN5 knockdown screening in A375 cells

**Source file:** 04_Figure3F_A375_NSUN5_knockdown_03.jpg

**File description:** loading-control or combined exposure

Source image 32. Preview of the accompanying source file; image content and aspect ratio are preserved.

# Source image 33

**Figure 3F**

NSUN5 knockdown screening in A375 cells

**Source file:** 04_Figure3F_A375_NSUN5_knockdown_04.tif

**File description:** loading-control or combined exposure

Source image 33. Preview of the accompanying source file; image content and aspect ratio are preserved.

# Source image 34

**Figure 3F**

NSUN5 knockdown screening in A375 cells

**Source file:** 04_Figure3F_A375_NSUN5_knockdown_05.tif

**File description:** loading-control or combined exposure

Source image 34. Preview of the accompanying source file; image content and aspect ratio are preserved.

# Source image 35

**Figure 3F**

NSUN5 knockdown screening in A375 cells

**Source file:** 04_Figure3F_A375_NSUN5_knockdown_06.tif

**File description:** loading-control or combined exposure

Source image 35. Preview of the accompanying source file; image content and aspect ratio are preserved.

# Source image 36

**Figure 3F**

NSUN5 knockdown screening in A2058 cells

**Source file:** 05_Figure3F_A2058_NSUN5_knockdown_01.tif

**File description:** loading-control or combined exposure

Source image 36. Preview of the accompanying source file; image content and aspect ratio are preserved.

# Source image 37

**Figure 3F**

NSUN5 knockdown screening in A2058 cells

**Source file:** 05_Figure3F_A2058_NSUN5_knockdown_02.tif

**File description:** NSUN5 target or combined exposure

Source image 37. Preview of the accompanying source file; image content and aspect ratio are preserved.

# Source image 38

**Figure 3F**

NSUN5 knockdown screening in A2058 cells

**Source file:** 05_Figure3F_A2058_NSUN5_knockdown_03.tif

**File description:** loading-control or combined exposure

Source image 38. Preview of the accompanying source file; image content and aspect ratio are preserved.

# Source image 39

**Figure 3F**

NSUN5 knockdown screening in A2058 cells

**Source file:** 05_Figure3F_A2058_NSUN5_knockdown_04.tif

**File description:** loading-control or combined exposure

Source image 39. Preview of the accompanying source file; image content and aspect ratio are preserved.

# Source image 40

**Figure 3F**

NSUN5 knockdown screening in A2058 cells

**Source file:** 05_Figure3F_A2058_NSUN5_knockdown_05.tif

**File description:** NSUN5 target or combined exposure

Source image 40. Preview of the accompanying source file; image content and aspect ratio are preserved.

# Source image 41

**Figure 3F**

NSUN5 knockdown screening in A2058 cells

**Source file:** 05_Figure3F_A2058_NSUN5_knockdown_06.tif

**File description:** NSUN5 target or combined exposure

Source image 41. Preview of the accompanying source file; image content and aspect ratio are preserved.

# Source image 42

**Figure 3H and Supplementary Figure S5C**

CENPF protein abundance after NSUN5 knockdown

**Source file:** 06_Figure3H_and_FigureS5C_CENPF_after_NSUN5_knockdown_01.tif

**File description:** CENPF target

Source image 42. Preview of the accompanying source file; image content and aspect ratio are preserved.

# Source image 43

**Figure 3H and Supplementary Figure S5C**

CENPF protein abundance after NSUN5 knockdown

**Source file:** 06_Figure3H_and_FigureS5C_CENPF_after_NSUN5_knockdown_02.tif

**File description:** HSP90 loading control

Source image 43. Preview of the accompanying source file; image content and aspect ratio are preserved.

# Source image 44

**Supplementary Figure S6A**

Exploratory YBX1 source blots after NSUN5 knockdown in A375 cells

**Source file:** 07_FigureS6A_A375_YBX1_after_NSUN5_knockdown_01.tif

**File description:** loading-control or combined exposure

Source image 44. Preview of the accompanying source file; image content and aspect ratio are preserved.

# Source image 45

**Supplementary Figure S6A**

Exploratory YBX1 source blots after NSUN5 knockdown in A375 cells

**Source file:** 07_FigureS6A_A375_YBX1_after_NSUN5_knockdown_02.tif

**File description:** YBX1 target or combined exposure

Source image 45. Preview of the accompanying source file; image content and aspect ratio are preserved.

# Source image 46

**Supplementary Figure S6A**

Exploratory YBX1 source blots after NSUN5 knockdown in A375 cells

**Source file:** 07_FigureS6A_A375_YBX1_after_NSUN5_knockdown_03.tif

**File description:** loading-control or combined exposure

Source image 46. Preview of the accompanying source file; image content and aspect ratio are preserved.

# Source image 47

**Supplementary Figure S6A**

Exploratory YBX1 source blots after NSUN5 knockdown in A375 cells

**Source file:** 07_FigureS6A_A375_YBX1_after_NSUN5_knockdown_04.tif

**File description:** loading-control or combined exposure

Source image 47. Preview of the accompanying source file; image content and aspect ratio are preserved.

# Source image 48

**Supplementary Figure S6B**

Exploratory YBX1 source blots after NSUN5 knockdown in A2058 cells

**Source file:** 08_FigureS6B_A2058_YBX1_after_NSUN5_knockdown_01.tif

**File description:** loading-control or combined exposure

Source image 48. Preview of the accompanying source file; image content and aspect ratio are preserved.

# Source image 49

**Supplementary Figure S6B**

Exploratory YBX1 source blots after NSUN5 knockdown in A2058 cells

**Source file:** 08_FigureS6B_A2058_YBX1_after_NSUN5_knockdown_02.tif

**File description:** NSUN5 target or combined exposure

Source image 49. Preview of the accompanying source file; image content and aspect ratio are preserved.
